# Gender-specific perception of job stressors and resources: a structural equation model-based secondary analysis

**DOI:** 10.3389/fpubh.2024.1463868

**Published:** 2024-12-13

**Authors:** Angelina Heub, Andrea Schaller, Martin Lange

**Affiliations:** ^1^Department of Fitness and Health, IST University of Applied Sciences, Düsseldorf, Germany; ^2^Department of Human Science, Institute of Sport Science, University of the Bundeswehr Munich, Neubiberg, Germany

**Keywords:** stress, gender, job demands, job resources, work, leadership, work time, co-worker support

## Abstract

**Objective:**

Stress is an extensive issue in modern society, affecting men and women differently. A better understanding of these patterns is required within the work context. Therefore, this study aimed to identify gender differences in the effects of stressors (quantitative demands, qualitative demands, working time) and resources (job control, quality of leadership, co-worker support) on subjective perceived stress across occupational groups.

**Methods:**

This study was conducted as a secondary data analysis based on the ‘German Study on Mental Health at Work’ data. The national representative cross-sectional sample included 4,118 employees. The data were analyzed using structural equation modeling.

**Results:**

Correlations between quantitative demands, working time, co-worker support, quality of leadership, job control, and subjective perceived stress were first confirmed for a total sample of employees. Gender differences in these interactions were then analyzed using multi-group equation modeling and a gender-stratified sample. Men and women showed an increase in subjective perceived stress for high quantitative demands. This increase was more prominent for men. Women further showed an increase in subjective perceived stress in response to long working time. High co-worker support, quality of leadership, and job control had stress-reducing effects but did not result in significant gender differences. No association was found between qualitative demands and subjective perceived stress.

**Conclusion:**

The results underline that not all working conditions significantly impact stress for both genders and gender differences exist only within the stressors. Hence, gendered strategies may only be required in some constellations. However, specific contexts require integrating gendered approaches in research and operational practice.

## 1 Introduction

### 1.1 Gender-specific prevalence and occurrence of stress at work

Stress can be understood as a significant disruption of physiological regulatory processes caused by internal or external stimuli that necessitate an adaptive response (1). Whether stress has a positive or negative character depends on the individual’s perception and interpretation of the situation (2). Sixty percent of Germans report exposure to stress (3), with 32% stating its impact on their daily lives (4). The workplace is one of the main sources of stress (5, 6), and employee stress levels have increased in recent years (6).

However, there are differences in the perception of stress among men and women. These gender differences require individual adjustments in the context of prevention (7) as well as a better understanding of the role of gender in the context of work (8). For instance, women experience less stress than men when receiving social support (9, 10). Further, studies highlight the importance of addressing these gender-specific effects regarding stress [e.g., European Agency for Safety and Health at Work (8) and Gilbert-Ouimet (11)]. To date, studies have shown gender differences in the effects of stressors and resources on stress for small constellations of demands and resources [e.g., Padkapayeva et al. (9), Vermeulen et al. (10), Rivera (12)], as well as in samples of specific occupational groups (13–15). However, the causes of stress are manifold, and the interaction of various factors is complex. The Job Demands-Resources (JD-R) Model (16, 17) offers an explanatory approach to the development of stress in the work context.

### 1.2 Job demands-resources model

The JD-R model provides a multidimensional framework to explain health and motivational processes. Health, motivational, and, indirectly, organizational outcomes are linked to the relationship between job demands and resources, which cluster the individual range of working conditions in an organization (18–21). Within the model, two main processes are delineated: (1) increased or inadequate job demands lead to a state of exhaustion and reduced health, and (2) a lack of or inadequate job resources reduces work-related motivation (17, 18, 22). Job demands are “physical, social, or organizational [stressors] of the job that require sustained physical or mental effort and are therefore associated with certain physiological and psychological costs” (17). Job resources represent physical, psychological, social, or organizational aspects that help achieve work goals, reduce job demands, and stimulate personal growth and development” (17).

Furthermore, an interaction between job demands and resources is described, influencing motivational and health-related follow-up processes (23). In this context, resources buffered job demands (24, 25). In addition, there are links between job demands and resources and organizational outcomes, mediated by reduced health and motivation (26, 27).

The JD-R model integrates stress and motivational theories and allows for a global application independent of specific demands, resources, or settings (16). From a cross-sectional perspective, examining the gendered effects of the multiple demand-and resource constellation prevalent in practice on the individual experience of stress remains open. To enhance comprehension of these gender differences, this research aims to examine how the stress level, as measured by *subjective perceived stress*, is affected by a multiple, cross-professional stressor and resource constellation in a gender-specific context. Specifically, this study aims to investigate gender differences in the effects of work stressors, including *quantitative demands*, *qualitative demands*, and *working time*, as well as the resources of *job control*, *co-worker support*, and *quality of leadership* on *subjective perceived stress* in a cross-professional setting.

## 2 Conceptual framework and hypothesis development

### 2.1 Relationship between job demands and stress

The existing body of research has well established that job demands can increase stress and therefore act as stressors in the context of work (15, 28–30). Many different working conditions are subsumed under the multidimensional construct of job demands. These can be divided into two major categories: quantitative and *qualitative demands*. *Quantitative demands* refer to the relationship between the number and amount of job requirements and the available time to handle them (31, 32). *Qualitative demands* describe the quality and complexity of a work task in relation to individual abilities and skills (e.g., intellectual or emotional competencies) (32, 33). These two basic dimensions do not represent the *working time*. However, the *working time* is relevant for almost all employees in all occupations (34) and is a critical factor in mental health (35). The *working time*, within this study, refers to the actual amount of time spent working rather than the contracted *working time*.

Associations between aspects of poor mental health, such as emotional exhaustion, depression, burnout, and stress-related disorders, and high job demands have been shown for the constructs of interest, *quantitative demands* (35–37); *qualitative demands* (38) and *working time* (39–41). Relationships with stress experience are apparent. Thus far, an increase in *quantitative demands* (38, 42) and *working time* (43–45) has been shown as potential risk factors for experiencing stress. Based on this knowledge, it is hypothesized that:

*H1: Quantitative demands* are positively related to *subjective perceived stress* in employees.
*H2: Working time* is positively related to employee s’ *subjective perceived stress*.

To the author’s knowledge, a relationship with stress has not yet been demonstrated for *qualitative demands* as an overall construct. However, associations with a reduced mental health status, depression, and burnout have been shown in a scoping review (38). Furthermore, relationships between increasing stress and high levels of cognitive demands (46), emotional demands (47), and complexity (48) were demonstrated. Based on these findings, it is postulated that:

*H3: Qualitative demands* are positively related to employees’ *subjective perceived stress*.

### 2.2 Relationship between job resources and stress

The buffering effect of job resources on stress has also been confirmed by several studies [e.g., Padkapayeva et al. (9), Xie et al. (15), and Huang et al. (49)]. Among the variety of job resources, some working conditions were found to be particularly important given their broad, cross-functional nature [cf. Morschhäuser et al. (34) and Rothe et al. (35)]. *Job control* is one of these resources. It refers to the influence that employees have over their work. This includes both the freedom to decide on the time and type of implementation of a work task (decision authority) as well as control over the use of concrete skills (skill discretion) (50). Furthermore, the resources of social support and *quality of leadership* are particularly important. Social support at work refers to how employees perceive the availability of *co-worker support* or support from direct supervisors when needed (31). It is important to note that within the scope of this study, social support from a supervisor constitutes a part of *quality of leadership*. However, leadership entails more than social support. According to Burr et al. (31), *quality of leadership* is concerned with “the next higher managers’ leadership in different contexts and domains”.

Again, associations with mental health have been well studied [e.g., Pohrt et al. (7), Brendel and Martus (51), Drössler et al. (52), and Rosen (53)]. Relationships between decreased stress levels and an adequate amount of resources were found when considering *co-worker support* and support from a supervisor (9, 30, 54); further aspects of employee leadership (55); as well as *job control* and related subscales, such as time flexibility or decision latitude (53, 56, 57). In line with these findings, the following hypotheses were formulated:

*H4: Co-worker support* is negatively related to employee s’ *subjective perceived stress*.
*H5: Quality of leadership* is negatively related to employee s’ *subjective perceived stress*.
*H6: Job control* is negatively related to *subjective perceived stress* in employees.

### 2.3 Gender differences in the impact of job stressors and resources on stress

Research has highlighted gender differences in the impact of various working conditions on stress development [e.g., Padkapayeva et al. (9), (10), Lian et al. (56), and Wang et al. (58)]. These differences can be attributed to dissimilarities in exposure to job demands and resources (59, 60). Furthermore, differing appraisals between males and females, resulting from biological differences, including hormonal or genetic factors (61), as well as cognitive and affective mechanisms (62), are a cause of varying stress levels.

About a gender-specific view of the resources included, Nieuwenhuijsen et al. (36) demonstrated in their systematic review that insufficient *co-worker support* is positively linked to stress-related disorders in men. For women, a correlation between *co-worker support* and stress-related disorders was unclear. However, Padkapayeva et al. (9) found no gender-related differences in the correlation between *co-worker support* and job-related stress. Furthermore, within the context of *quality of leadership*, appreciation from supervisors has been proven to be more predictive of depressive symptoms for women (7). In contrast, social support from supervisors has only been found to reduce stress-related disorders for men. Effects of *job control* on stress have so far been controversial concerning gender [cf. Padkapayeva et al. (9), Steptoe and Willemsen (57), De Bruin and Taylor (63), Melin et al. (64)].

**Figure 1:**
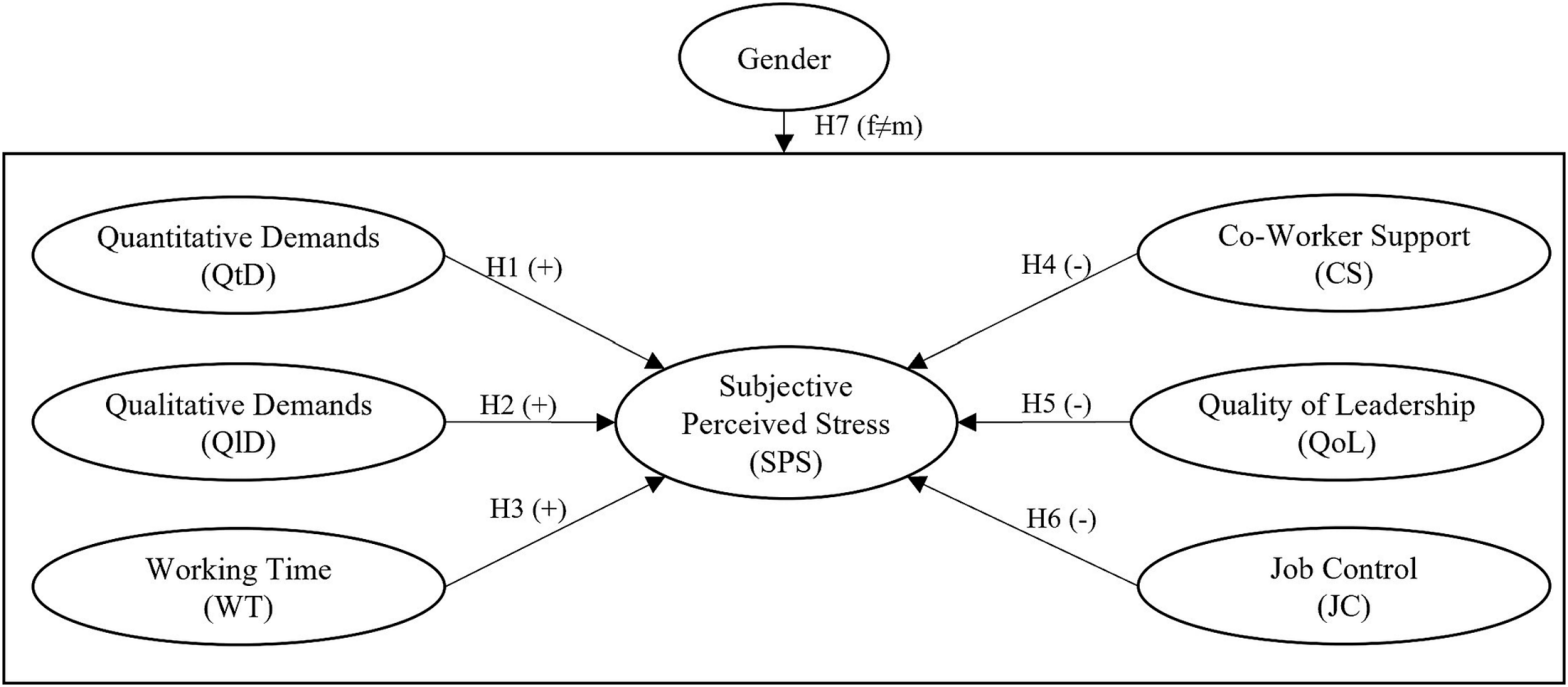
Hypothetical model of the gender-specific impact of job demands and resources on perceived stress.

The gendered effects of stressors have been studied mainly in the context of psychological constructs but rarely specifically to stress. *Quantitative demands* have proven to be more critical for men compared to women regarding mental illness (12, 65, 66). An association between *qualitative demands* and mental illness was only observed in women (12). In contrast, separate elements of *quantitative demands* and *qualitative demands*, such as working quickly or performing complex tasks, have already been considered concerning stress in the context of gender-specificity. *Quantitative demands* have proven to be more relevant predictor for men compared to women regarding distress (10). Sub-aspects of both constructs were found to be more important for women predicting stress (48). Extended *working time* also increases the risk of depression and burnout, particularly for women (67, 68). The impact on men remains unclear, as studies have yielded conflicting results [cf. Choi et al. (67), Hu et al. (68), and Weston et al. (69)]. Even if the exact impact patterns remain unclear in some cases, gender-specific differences have been identified. Summarizing the previous findings, it is hypothesized that:

*H7:* The relationships between *quantitative demands*, *qualitative demands*, *working time*, *co-worker support*, *quality of leadership*, *job control*, and *subjective perceived stress* significantly vary between males and females. Figure 1 summarizes the postulated hypotheses in a hypothetical framework.

## 3 Methodology

### 3.1 Study design

The present study was performed as secondary research using a quantitative approach based on data from the “German Study on Mental Health at Work” (S-MGA). This approach allows the hypothetical model to be tested on a representative sample of all occupational groups in Germany. The implementation was carried out according to the STROSA-2 standards (Standardized Reporting Routine for Secondary Data Analyses; see Supplementary file) (70).

### 3.2 Data source

The S-MGA (71), founded by the BAuA (Bundesanstalt für Arbeitsschutz und Arbeitsmedizin), is a cohort survey aimed at offering representative information on the workability and health of German employees. Employees with social insurance who were born between 1951 and 1980 were included in the survey. Sampling was carried out using a two-stage stratified random approach. Computer-assisted personal interviews with trained interviewers and paper and pencil questionnaires were used for data collection (72). The S-MGA contains information from two survey periods on workability, functional and mental health, main employment status, secondary employment, and sick leave (73). Baseline data from November 2011 to June 2012 were used for the underlying sample. This database contained 4,511 valid interviews (response rate: 35.7%) from a representative cross-sectional sample of the German working population.

### 3.3 Data flow

The Institute for Applied Social Sciences (infas) collected and prepared data. Direct identifiers were removed from the data before transmission to the BAuA (72). Security strategies were implemented to ensure maximum anonymity. The dataset did not contain sensitive characteristics (72). Data access to the Scientific User File provided by the FDZ-BAuA was enabled via a data exchange server. A data use agreement governed the provision and use of secondary data. Data were *de facto* anonymized when transferred. Data protection was ensured according to the requirements of the General Data Protection Regulation (74).

### 3.4 Selection criteria and units of analysis

For the underlying sample, only employees in a dependent professional relationship who reported having a supervisor, clearly defined working hours, and provided information on their gender were considered. Gender differences were identified by comparing indices that are highly sensitive to sample size, so the samples of males and females were equalized. A random sample of *n* = 2,059 was drawn from the male dataset to avoid bias due to sample size (75, 76). Survey data from the employees subject to social insurance as of December 31, 2010, and born between 1951 and 1980, which corresponded to the selection criteria described above, were analyzed. For the gender-specific analysis, participants were grouped into males and females.

### 3.5 Measures

Information on gender was obtained from employment histories held by the Institute for Employment Research and verified during interviews with participants. Items from the German version of the Copenhagen Psychosocial Questionnaire (COPSOQ) (77) were used to assess *quantitative demands*, *quality of leadership*, *co-worker support*, and *job control*. All items were measured using 5-point Likert scales (e.g., always, often, sometimes, rarely, never/hardly ever). All other items were carefully selected using the top-down technique (78) by established questionnaires, scientific findings, and occupational psychology models [cf. MacKenzie et al. (79) and Weiber and Sarstedt (80)]. The variables *qualitative demands* and *working time* were each represented by global indicators using self-rated single-item measures [cf. Pattloch et al. (72)]. Both were treated as ordinal data. *Working time* was assessed by the number of hours worked per week and then grouped into seven categories with similar intervals.

In line with the procedure described above, *subjective perceived stress* was operationalized according to the cognitive-emotional level of the stress response. A total of four items from the “Scale of Positive and Negative Experience” (81), the “Psychological Well-Being Scale” (82), and the “Short-Form Health Survey” (83, 84) were used for this purpose. Items were scored on 5-to 7-point rating scales. Detailed information on the items used can be found in Supplementary file.

### 3.6 Covariates

To control for confounding variables, factors that influence the perception of stress were also considered (63, 85). For practical purposes and to avoid the risk of misinterpretation, we controlled for the most relevant demographic categories (63). Thus, age of the participants at the time of data collection (86, 87), educational level (88, 89), income (88), children under 14 years in the household (90), caregiving (91), and hours worked in paid second jobs (92, 93) were included as confounders (see Supplementary file).

### 3.7 Statistical analysis

Structural equation modeling (SEM) was used as the primary analytical approach to analyze the interdependencies of multiple manifest and latent variables (80, 94). To examine disparities between genders, the underlying framework of the impact of stressors and resources on *subjective perceived stress* was first validated within a sample that included both males and females. Subsequently, within this framework, gender differences were assessed using a stratified sample. An *a priori* power analysis was conducted to determine the minimum sample size required for testing the hypothesized equation model using “*A-priori* Sample Size Calculator for Structural Equation Models” (95). The sample size required to achieve 80% power to identify small effects, with a significance level of *α* = 0.05, was *n* = 1,808. Hence, the sample sizes of *n* = 4,118 for the entire sample and *n* = 2,059 for the samples stratified by gender are valid to test the hypothesized equation model. Differences between these two groups were analyzed with the Mann–Whitney-*U*-test (96).

Data management and hypothesis testing were performed using SPSS version 29.0 and IBM SPSS AMOS 29 (Statistical Package for the Social Science, Analysis of Moment Structures). Outliers were identified as plausible and retained to avoid manipulation of results (97). Univariate non-normality was indicated by values outside the cut-offs of ±2 for skewness, ± 7 for kurtosis, and critical ratios (C.R.) > | 1.96 | (98, 99). The analyzed data contained 1,663 missing values (1.50%) (Supplement C). The missing mechanism was assumed to be missing at random (MAR) using Little’s missing completely at random (MCAR) test (100), visual inspection of missing data patterns (94), and rational reflection consideration (101, 102).

For SEM, a two-stage modeling approach was used (103). First, the measurement model was assessed. Principal component analysis (PCA) and confirmatory factor analysis (CFA) were therefore carried out for the entire study sample and, regarding multi-group analysis, for each gender separately (80). Recursive models were then calculated using covariance-based single-group and multi-group SEM. The models were estimated using maximum likelihood estimation (ML) and bootstrapping with 5,000 iterations to address non-normality and ordinal data (99, 104). Model fits were assessed using Root Mean Square Error of Approximation (RMSEA), Standardized Root Mean Squared Residual (SRMR), Comparative Fit Index (CFI), and Adjusted-Goodness-of-Fit-Index (AGFI). CFI and AGFI were assumed to be ≥0.90 (80). For RMSEA and SRMR, values ≤0.08 were considered acceptable (105). Following Schreiber (106), chi-square and degrees of freedom were provided for better interpretation of the values based on them but owing to the sensitivity of chi-square to sample size, they were not included in the direct assessment for model fit.

## 4 Results

### 4.1 Selection of study population and descriptive results

**Figure 2:**
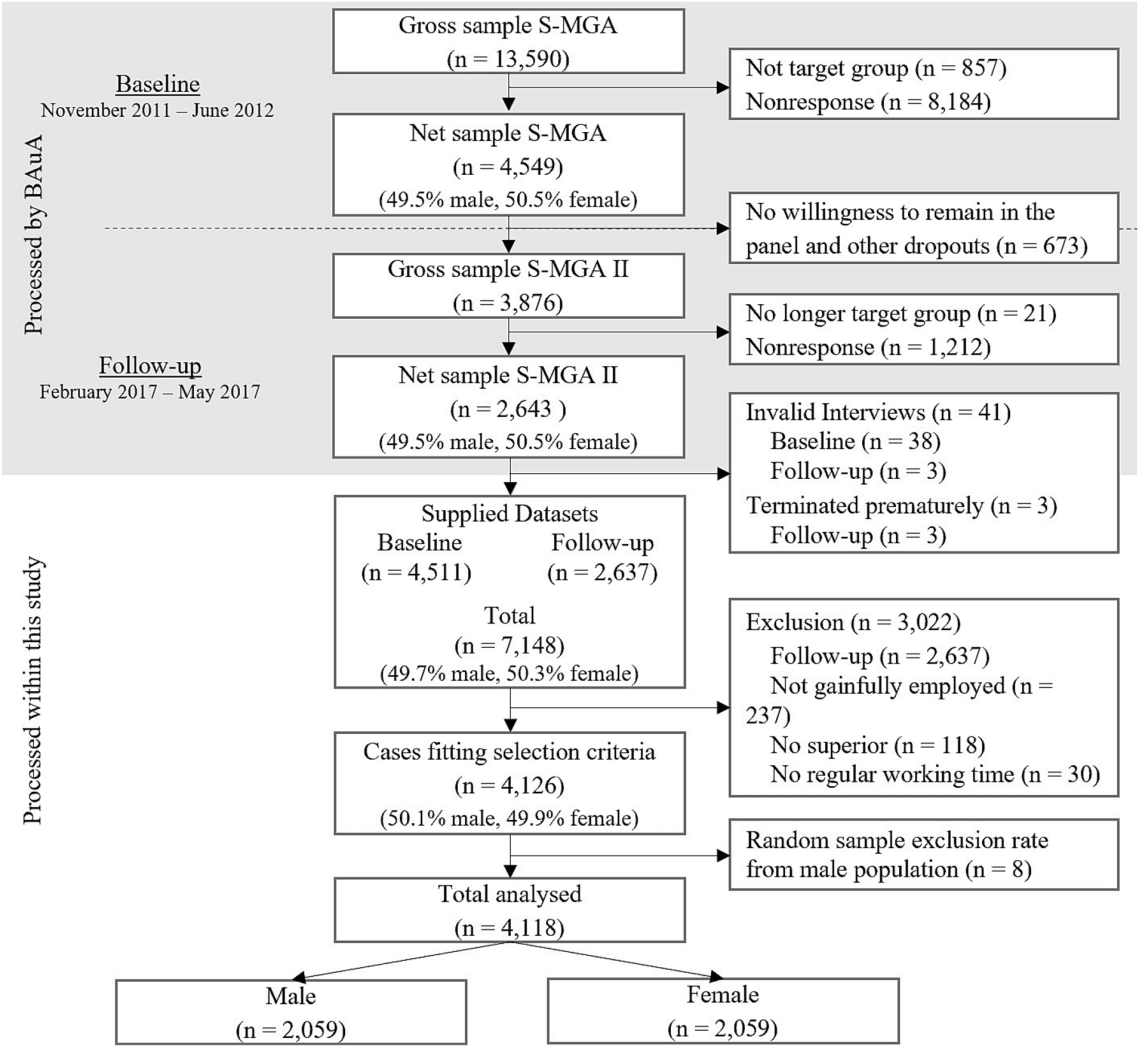
Flowchart of selection process from original to study population.

From the provided sample of *n* = 7,148 with data from two survey waves, data from *n* = 4,118 employees were used for the current study. The entire sampling and selection process is shown in Figure 2. The final sample included 2,059 women and men each, from different occupational groups and companies covered by social security. With a median age range of 45–49 years for both males and females, the age span varied from 31 to 60 years. Approximately 72% of male and 70% of female respondents were married or in a civil partnership, and 69% of each gender lived in a household without children. The majority of respondents (male: 57.50%, female: 61.83%) had received vocational education or training, while over a third (male: 37.73%, female: 32.69%) had completed higher education (e.g., university degree). The vast majority of men worked full-time (93.88%), whereas roughly half of women were employed full-time (49.34%). Approximately 10% of both males and females reported having a second job.

Descriptive statistics on the items of the main study variables for the total sample and samples stratified by gender are presented in Table 1.

**Table 1:** Descriptive statistics of the total sample and the sample stratified by gender for key items.

Items	Averages (SD)^a^			Theoretical range	*p-*value for gender differences^b^
	Total (*n* = 4,118)	Male (*n* = 2,059)	Female (*n* = 2,059)		
Stressors					
QtD_1	2.67 (1.15)	2.66 (1.13)	2.68 (1.17)	1–5	0.570
QtD_2	3.00 (1.10)	3.06 (1.08)	2.94 (1.11)	1–5	<0.001^***^
QtD_3	2.93 (1.15)	2.93 (1.12)	2.93 (1.17)	1–5	0.928
QtD_4	2.33 (1.08)	2.38 (1.06)	2.28 (1.11)	1–5	<0.001^***^
QlD_1	2.00	2.00	2.00	1–4	<0.001^***^
WT_1	5.00	5.00	3.00	1–8	<0.001^***^
Resources					
JC_1	2.95 (1.44)	3.05 (1.42)	2.84 (1.45)	1–5	<0.001^***^
JC_2	2.12 (1.34)	2.30 (1.37)	1.94 (1.28)	1–5	<0.001^***^
JC_3	2.08 (1.22)	2.14 (1.21)	2.02 (1.23)	1–5	<0.001^***^
QoL_1	3.23 (1.15)	3.18 (1.12)	3.29 (1.18)	1–5	<0.001^***^
QoL_2	3.48 (1.08)	3.42 (1.06)	3.54 (1.10)	1–5	<0.001^***^
QoL_3	3.25 (1.11)	3.18 (1.10)	3.31 (1.10)	1–5	<0.001^***^
QoL_4	3.21 (1.12)	3.22 (1.08)	3.20 (1.15)	1–5	0.755
CS_1	3.71 (1.19)	3.77 (1.12)	3.65 (1.25)	1–5	0.065
CS_2	3.69 (1.21)	3.69 (1.18)	3.70 (1.25)	1–5	0.323
CS_3	2.93 (1.21)	2.98 (1.19)	2.87 (1.23)	1–5	0.008^**^
SPS					
SPS_1	2.32 (0.79)	2.35 (0.79)	2.30 (0.79)	1–5	0.097
SPS_2	2.60 (1.21)	2.64 (1.26)	2.56 (1.16)	1–7	0.185
SPS_3	2.73 (0.81)	2.72 (0.80)	2.75 (0.81)	1–5	0.171
SPS_4	2.59 (0.85)	2.51 (0.84)	2.68 (0.85)	1–5	<0.001^***^

QtD, quantitative demands; QlD, qualitative demands; WT, working time; CS, co-worker support; QoL, quality of leadership; JC, job control; SPS, subjective perceived stress; ^a^Means indicate the average for metric items, median was calculated for ordinal items. SD values were computed for metric items only; ^b^Differences were evaluated using the Mann–Whitney-*U*-test; **p < 0.01; ***p < 0.001.

### 4.2 Main results

#### 4.2.1 Measurement model

Five reflective constructs (*quantitative demands*, *job control*, *quality of leadership*, *co-worker support*, and *subjective perceived stress*) were assessed using multi-item scales. For all groups (total sample, male and female), the PCA confirmed the structure of the measurement models (see Supplementary file). CFA showed good fits in all groups (total: *χ*^2^ = 1437.76, df = 237, CFI = 0.96, RMSEA = 0.04, SRMR = 0.03, AGFI = 0.96, male: *χ*^2^ = 891.38, df = 237, CFI = 0.96, RMSEA = 0.04, SRMR = 04, AGFI = 0.95, female: *χ*^2^ = 810.27, df = 237, CFI = 0.97, RMSEA = 0.03, SRMR = 0.03, AGFI = 0.95). Significant factor loadings were found for all indicators. Within the total sample, 15 out of 18 indicators achieved good indicator reliability [squared multiple correlation (SMC) ≥ 0.40 (80); see Supplementary file]. Reliability and convergent validity values for the multi-item constructs are summarized in Table 2.

**Table 2:** Reliability and convergent validity of the multi-item constructs of the measurement model.

Construct	Total (*n* = 4,118)			Male (*n* = 2,059)			Female (*n* = 2,059)		
	*α*	CR	AVE	*α*	CR	AVE	*α*	CR	AVE
QtD	0.84	0.84	0.57	0.84	0.84	0.57	0.83	0.84	0.57
CS	0.75	0.76	0.52	0.72	0.73	0.47	0.78	0.80	0.57
QoL	0.85	0.85	0.58	0.83	0.83	0.55	0.87	0.87	0.62
JC	0.68	0.68	0.42	0.68	0.68	0.41	0.68	0.68	0.42
SPS	0.75	0.75	0.43	0.74	0.74	0.42	0.75	0.76	0.44

QtD, quantitative demands; CS, co-worker support; QoL, quality of leadership; JC, job control; SPS, subjective perceived stress; *α*, standardized cronbach’s alpha; CR, composite Reliability; AVE, average variance extracted.

Regarding the composite reliability (CR), all groups showed values above the threshold of 0.60 (107) for all constructs. Furthermore, good to acceptable values for the average variance extracted (AVE) could be demonstrated. A value of ≥0.50 was assumed to indicate good convergence validity (108). Cronbach’s alpha was computed to evaluate internal consistency reliability. *Quantitative demands*, *co-worker support*, *quality of leadership*, and *subjective perceived stress* achieved good reliability. *Job control* fell below the threshold of *α* ≥ 0.70 (109) in all groups. Discriminant validity was confirmed using the Heterotrait-Monotrait Ratio of Correlations (HTMT). For all groups, the HTMT values were below the critical threshold of 0.85 [(110); see Supplementary file].

Measurement invariance was tested across genders. An acceptable fit of the unconstrained model (M^U^) (*χ*^2^ = 1704.79, df = 475, CFI = 0.96, RMSEA = 0.03, SRMR = 0.04, AGFI = 0.95), significantly non-zero factor loadings, and factor correlations <1 confirmed configural invariance. Metric invariance was proven by comparison of the fit of the M^U^ with the measurement weights model (M^M^) (i.e., constrained factor loadings) (ΔCFI = 0.00, ΔRMSEA = 0.00, ΔSRMR = 0.00). Since the relationship structures, but not the mean values of the constructs, were compared between the groups within multi-group SEM, scalar invariance was not tested (111). Overall, the results confirm the validity of the measurement model in both groups, males and females (see Supplementary file).

#### 4.2.2 Structural equation model

##### 4.2.2.1 Effects of stressors and resources on subjective perceived stress

Before testing for gender differences, the underlying framework was considered. In order to assess the hypothesized general effects of the stressors (*quantitative demands*, *qualitative demands*, and *working time*) and resources (*co-worker support*, *quality of leadership*, and *job control*) on *subjective perceived stress*, SEM was used. The equation model (see Supplementary file) was therefore based on the entire sample. Table 3 summarizes the SEM results without any Heywood cases having occurred.

**Table 3:** SEM analysis of the effects of job demands and resources on *subjective perceived stress* for a total sample of employees.

Hypothesized relationships	Unstandardized estimates	Standardized estimates	S.E.	C.R.	*p-*values	Hypothesis supported
**H1:** QtD ^+^_➔_ SPS	0.27	0.30	0.02	14.47	<0.001^***^	Supported
**H2**: QlD ^+^_➔_ SPS	−0.03	−0.04	0.02	−1.76	0.079	Not supported
**H3**: WT ^+^_➔_ SPS	0.05	0.09	0.02	4.07	<0.001^***^	Supported
**H4**: CS ^−^_➔_ SPS	−0.07	−0.09	0.02	−4.71	<0.001^***^	Supported
**H5**: QoL ^−^_➔_ SPS	−0.20	−0.28	0.02	−13.99	<0.001^***^	Supported
**H6**: JC ^−^_➔_ SPS	−0.09	−0.12	0.02	−5.68	<0.001^***^	Supported

*n* = 4,118. QtD, quantitative demands; QlD, qualitative demands; WT, working time; CS, co-worker support; QoL, quality of leadership; JC, job control; SPS, subjective perceived stress; S.E., standard error; C.R., critical ratio; Adjusted for age, educational level, income, children under 14 years in the household, caregiving, hours worked in paid second jobs. Estimation method: ML; Model fit statistics: *χ*^2^ = 3434.92, df = 249, CFI = 0.90, RMSEA = 0.06, SRMR = 0.086, AGFI = 0.91; ***p < 0.001.

The model was identified and indicated an acceptable fit (*χ*^2^ = 3434.92, df = 249, CFI = 0.90, RMSEA = 0.06, SRMR = 0.086, AGFI = 0.91). Considering the adjusted beta estimates, significant positive associations on *subjective perceived stress* were shown for *quantitative demands* (*β* = 0.30, *p* < 0.001) and *working time* (*β* = 0.09, *p* < 0.001). According to the hypotheses, demanding stressors *quantitative demands* and *working time* were associated with an increase in *subjective perceived stress*. The estimates for *qualitative demands* turned out to be insignificant and contrary to the expected direction of effect. Significant negative associations on *subjective perceived stress* were found for the resources *co-worker support* (*β* = −0.09, *p* = < 0.001), *quality of leadership* (*β* = −0.28, *p* < 0.001), and *job control* (*β* = −0.12, *p* < 0.001). Therefore, it can be concluded that reduced *subjective perceived stress* is associated with higher levels of *co-worker support*, *quality of leadership*, and *job control* resources. *Quantitative demands* had the strongest association with *subjective perceived stress*. *Quality of leadership* turned out to be the most effective resource. In total, 21.43% of the variance in *subjective perceived stress* could be explained by the working conditions examined.

##### 4.2.2.2 Gender differences within the hypothesized framework

With the aim of assessing gender differences in stress perceptions of job stressors and resources, multi-group SEM was used. First, the overall difference for the hypothesized model was tested across genders. Structural invariance across males and females was assessed. Therefore, a model with equally constrained factor loadings and path coefficients was compared with the M^U^. While the chi-square difference test was found to be significant (Δχ^2^ = 34.21, *p* = 0.017), comparison of the absolute and incremental fit indices showed no discrepancies between genders (ΔCFI = 0.00, ΔRMSEA = 0.00, ΔSRMR = 0.00, cf. Supplementary file). Nonetheless, as the chi-square difference test is commonly used as a single indicator to test for invariance, a significant difference within the considered framework between males and females can be assumed.

To identify where gender differences manifest themselves in the model, the individual paths from the stressors and resources to *subjective perceived stress* were considered for each gender. This was done by constraining the unstandardized estimates separately (137). Table 4 shows the path coefficients and their critical ratios for males and females, along with the group difference test results. Detailed information on SEM for males and females are accessible in supplement H. The multi-group model was identified and showed an acceptable fit (*χ*^2^ = 3648.82, df = 498, CFI = 0.90, RMSEA = 0.04, SRMR = 0.09, AGFI = 0.90). The squared multiple correlations for *subjective perceived stress* were *R*^2^ = 0.25 in males and *R*^2^ = 0.19 in females. The gender-specific analysis of the effect structure showed slight differences between genders. A significant increase in *subjective perceived stress* was found for *working time* in women (*β* = 0.06, *p* < 0.001), whereas no significant relationship was found in men (*β* = 0.01, *p* = 0.588). For both genders, *co-worker support*, *quality of leadership*, and *job control* were found to have a significant negative association on *subjective perceived stress*, whereas *quantitative demands* had a significant positive effect. The strength of the association of *job control* with *subjective perceived stress* did not differ between genders (*β*_male/female_ = −0.09, *p*_male/female_ < 0.001). For *quantitative demands* (β_male_ = 0.33, *p*_male_ < 0.001; *β*_female_ = 0.23, *p*_female_ < 0.001), *co-worker support* (*β*_male_ = −0.10, *p*_male_ < 0.001; *β*_female_ = −0.05, *p*_female_ = 0.008), and *quality of leadership* (*β*_male_ = −0.21, *p*_male_ < 0.001; *β*_female_ = −0.20, *p*_female_ = 0.007), *β*-values indicated a greater association with *subjective perceived stress* for men. However, the only significant gender difference in the strength of association was found for *quantitative demands* (Δ*χ*^2^ = 5.93, *p* = 0.015). Accordingly, high levels of *co-worker support*, *quality of leadership*, and *job control* are equally associated with a decrease in *subjective perceived stress* among both males and females. High *quantitative demands* were associated with an increase in *subjective perceived stress*. The increase was found to be greater in men. Like the results for the total sample, for both genders, *quantitative demands* had the strongest association with *subjective perceived stress*, and *quality of leadership* was found to be the most relevant resource. No significant association of *qualitative demands* with *subjective perceived stress* was found for either gender.

**Table 4:** Multi-group SEM analysis results for males and females.

Hypothesized relationships	Male	Female	Group Differences	
	Unstandardized estimates (C.R.)	Unstandardized estimates (C.R.)	Δ*χ*^2^ (Δdf)^a^	*p*-value for difference^b^
QtD ^+^_➔_ SPS	0.33^***^ (11.46)	0.23^***^ (8.52)	5.93 (1)	0.015^*^
QlD ^+^_➔_ SPS	−0.01 (−0.42)	−0.04 (−2.04)	0.64 (1)	0.423
WT ^+^_➔_ SPS	0.01 (0.54)	0.06^***^ (3.21)	3.55 (1)	0.059
CS ^−^_➔_ SPS	−0.10^***^ (−4.18)	−0.05^**^ (−2.70)	2.99 (1)	0.084
QoL ^−^_➔_ SPS	−0.21^***^ (−9.35)	−0.20^***^ (−10.18)	0.02 (1)	0.898
JC ^−^_➔_ SPS	−0.09^***^ (−3.65)	−0.09^***^ (−4.00)	0.02 (1)	0.878

Male and female sample each *n* = 2059. QtD, quantitative demands; QlD, qualitative demands; WT, working time; CS, co-worker support; QoL, quality of leadership; JC, job control; SPS, subjective perceived stress; C.R., critical ratio; *χ*^2^, chi-square value, df, degrees of freedom. Adjusted for age, educational level, income, children under 14 years in the household, caregiving, hours worked in paid second jobs. Estimation method: ML; ^a^Differences between unconstrained model and model constrained by the relationship under consideration; ^b^*p*-value for chi-square differences across men and women when the relationship under consideration is constrained; Model fit statistics: *χ*^2^ = 3648.82, df = 498, CFI = 0.90, RMSEA = 0.04, SRMR = 0.09, AGFI = 0.90; *p < 0.05. **p < 0.01. ***p < 0.001.

## 5 Discussion

### 5.1 Main results

Scientific evidence highlights the importance of addressing gender differences in the perception of work-related stress [e.g., European Agency for Safety and Health at Work (8) and Gilbert-Ouimet et al. (11)]. The purpose of this study was to examine gender-specific differences in the effect of job stressors and resources on *subjective perceived stress*.

The overall examination of the hypothesized model, which was conducted prior to the analysis of gender differences, considering men and women simultaneously, demonstrated that high levels of *quantitative demands* and *working time* are associated with an increase in *subjective perceived stress*, while high *co-worker support*, *quality of leadership*, and *job control* are linked to a reduction in *subjective perceived stress*. *Qualitative demands* do not significantly impact *subjective perceived stress*. Consequently, except for hypothesis 2, these outcomes confirm hypothesis 1 and hypotheses 3–6. Hypothesized gender differences within this framework are evident for the framework as a whole, but not for each impact of stressors and resources on *subjective perceived stress* in detail. The effect of *working time* on *subjective perceived stress* was found to be relevant only for women, and high *quantitative demands* were associated with an increase in *subjective perceived stress* for both genders, but more so for men. No differences in either relevance or strength of effect could be demonstrated for *qualitative demands*, *co-worker support*, *quality of leadership*, and *job control*. Therefore, H7 must be rejected.

### 5.2 Interpretation

#### 5.2.1 Impact of stressors on stress perception and gender differences within this relationship

Considering males and females simultaneously, this research confirms previous studies that found associations between increased stressors of *quantitative demands* (38, 42) and *working time* (43, 45) and an increase in stress.

Against the background of gender-specificity in line with Rivera-Torres et al. (12), the results regarding *quantitative demands* show an increase in stress for both genders when demands are high. This effect is stronger for men than for women. In contrast, Herrero et al. (48) found a greater hazard for women, while Rivera-Torres et al. (12) found no differences for either gender. Overall, these findings are controversial so far. However, it should be mentioned that, in contrast to the present study, Herrero et al. (48) only considered partial aspects of *quantitative demands* such as tight deadlines or fast work, and Rivera-Torres et al. (12) operationalized stress via the risk of illness or accidents. One possible explanation for the stronger impact on men within this study may be attributed to the different distribution of stressors between the genders. Overall, the proportion of *subjective perceived stress* explained by the included predictors is greater for men than for women. This indicates that, especially for females, a considerable number of predictors can be found outside the work environment (58).

Gender-specific analysis of *working time* revealed that *subjective perceived stress* was impacted by *working time* only in women. These findings resemble research on burnout and depression, which suggests that *working time* is more important for women (68, 69). One potential explanation for this phenomenon is that women tend to have more responsibilities outside of work, such as caring for their household or family (112). Increased *working time* may limit the time they have available to meet these obligations, resulting in heightened demands and ultimately more stress. However, the length of *working time* also affects the duration for which employees are exposed to further job demands (113). Thus, although no evidence was found for direct effects in men within this study, an indirect influence of stress through *working time* is conceivable. Investigating this impact is beyond the scope of this study and may be considered in future research.

The effects of *qualitative demands* on *subjective perceived stress* were negative and insignificant among employees in general and when considering gender individually. These findings contradict previous research that has linked high *qualitative demands* at least to a decrease in mental health (38) and identified subscales of *qualitative demands* that correlate with stress (46–48). Furthermore, in terms of gender-specificity, Rivera-Torres et al. (12) found *qualitative demands* to be relevant in reducing stress for women, which contradicts these studies findings but also no significant effect for men. Overall, the effects of this study might be biased by the operationalization of *qualitative demands*. Due to the secondary data, only the complexity of the job was measured. Individual requirements of the respondents could not be taken into account, although these would also have been relevant for classification as a stressor (17, 114). Interpretation of the results on *qualitative demands* is therefore very limited. Nonetheless, the results cannot confirm that *qualitative demands*, measured by complexity, act as a stressor within the underlying framework. Further research is needed to determine whether *qualitative demands* as an overall construct is actually not related to stress or whether the lack of significance here is due to gaps in the operationalization of rather *qualitative demands* or *subjective perceived stress*.

#### 5.2.2 Impact of resources on stress perception and gender differences within this relationship

For the resources included, a holistic consideration of men and women is also in line with the existing literature, as a reduction in stress is associated with a sufficient extent of *co-worker support* (9, 30, 115), *quality of leadership* (55), and *job control* (53, 57). Gendered analysis shows that these effects are equally evident for males and females. Findings on *job control* and *co-worker support* are therefore consistent with previous studies (9, 57, 64). Results on *quality of leadership* offer additional clarity into the understudied gendered relationship between *quality of leadership* as an overall construct and *subjective perceived stress*. Based on the results, it can be assumed that *quality of leadership*, as opposed to social support from the supervisor, is associated with lower *subjective perceived stress* for both men and women. This finding needs to be verified in further studies.

One finding that stands out from most of the previously reported results is the gender equality of the effect of resources on *subjective perceived stress*. However, the results are consistent with those of Xie et al. (15), who found no moderating effect of gender on the relation between resources and stress in a sample of social workers. In this context, Felsten (116) suggested that societal developments may lead to a reduction of gender-specific differences in stress levels over time. It is possible that this trend is reflected in the results of this study and explains the lack of differences in resources. The persistence of gendered differences in stressors may reflect biological differences of sex that remain unaffected by socially induced changes. Job characteristics and gender-based approaches offer another explanation for stressor differences prior to Felsten’s (116) theory. There is a correlation between gender differences in health and gender differences in exposure to job characteristics (117). Thus, it is possible that recent efforts to achieve gender equality in the workplace have achieved equality in the distribution of resources. In contrast, stressors might not yet have been equalized to the same extent and result in a still significant gender gap.

An alternative explanation for gender equality in the effects resources is Hyde’s (118) gender similarity hypothesis, which argues that men and women do not differ meaningfully on various psychological constructs. Considering the existing, but very small, gender differences in the effects of stressors on *subjective perceived stress* within this study, Hyde’s theory also seems noteworthy.

However, as previously noted, these theories contradict many studies that have been able to demonstrate gender differences within resources. Therefore, it is crucial to clarify whether the current state of research is affected by publication bias. Future research on this topic is highly recommended.

Overall, supporting previous research, gender differences in the relationship between working conditions and stress could be demonstrated (9, 12, 56, 58, 69). However, a more nuanced examination of individual effects reveals that these cannot be generalized to all working conditions.

#### 5.2.3 Implications for addressing gender in the context of job stressors, resources, and stress

It can be concluded that gender differences are still relevant, although they may be diminishing, and should be taken into account in future studies as well as in organizational practice. In the research context, but also in analyses in the operational context, samples stratified by gender should be preferred or at least considered as a complementary measure, if possible, and if anonymity can be guaranteed. Otherwise, relevant effects may remain undetected.

With regard to stress-reducing interventions in the operational setting, the importance of gender is initially secondary, however not irrelevant. Initial efforts to prevent and reduce stress can be made without addressing men and women separately. Therefore, *quantitative demands* and *quality of leadership* have been proven to be the most impactful factors in terms of *subjective perceived stress* for both genders. At least in the German labor market, these can be starting points for positively counteracting stress and its long-term consequences in a workplace setting. However, these findings do not imply that gendered approaches related to *quantitative demands* and *quality of leadership* should be principally disregarded. Rather, it is offered an opportunity to allocate limited resources onto interventions that are equally accessible to both males and females. Furthermore, small effect sizes among the other relevant stressors and resources do not mean that they are irrelevant. The phenomenon of rather small to moderate effects is also known from other studies in this context and seems quite plausible, especially with regard to the multidimensional outcome (119). Rothe et al. (35) explicitly warn against underestimating small effect sizes of working conditions that are relevant for a large number of employees, as is the case here. Thus, further approaches beyond the two most important variables identified may be useful. Gender-specific strategies are suggested for follow-up interventions that address the *working time*. This allows for a more focused allocation of resources and increases the effectiveness of interventions. Furthermore, especially for women, interventions that go beyond the design of the immediate working conditions may be effective (e.g., reconciliation of work and family). Interventions to reduce stress through enhancing *co-worker support* or *job control* do not require separate strategies for men and women.

Finally, it should be noted that regardless of the working condition that is being optimized, it may be worth considering both genders in an entire sample if there are not enough employees or participants to conduct stratified analyses or interventions. Moreover, if the target group has already been classified according to other variables, such as age, further differentiation may not be meaningful.

### 5.3 Internal validity and risk of bias

The above conclusions need to be considered in the light of some methodological issues. Due to the research design, it was not possible to control for confounding during data collection. The influence of response bias, especially social desirability, cannot be excluded given the sensitive nature of the study. However, the avoidance of interviewers and the use of questionnaires in appropriate subject areas minimize bias (73). Interviewer training was used to reduce interviewer bias and to ensure high data quality (73, 120). Though, the evaluation of working conditions was based on respondents’ self-assessments. It therefore does not provide an objective assessment of the actual working situation. Thus, reverse causality cannot be ruled out, particularly in relation to mental health and perceived stressors (121). In terms of representativeness, the sample is unbiased. Despite a response rate of around 36%, the impact of non-response bias is considered low based on a selectivity analysis (73). Selection bias is not expected due to a carefully conducted sampling process by the BAuA.

Regarding the constructs of interest, it should first be noted that the operationalization was limited by the database. Most variables could be measured using the COPSOQ as a valid and established instrument (77). However, *job control* failed to achieve internal consistency reliability. Moreover, well-established questionnaire scales were not available for all constructs. A careful conceptualization of *subjective perceived stress*, based on scientific evidence, nevertheless allows for adequate quality. Content validity can be presumed (80). However, no additional expert validation was carried out. In addition, in two cases, single items had to be used. For that reason, the measurement of *qualitative demands* is accompanied by a reduced information content (122). Given the lack of external criteria, criterion validity was not verified. To assess construct validity, nomological and discriminant validity were considered. As the SEM model fit confirms the underlying relationship between the constructs, nomological validity is concluded (123). HTMT values confirm discriminant validity (110). An assessment of convergent validity as a further component of construct validity was not possible as no additional measurement procedures were available. However, the AVE values do not indicate a lack of convergent validity (108). Overall, construct validity can be assumed with caution, as convergent validity cannot be definitively assessed.

Further to declare is that both independent and dependent variables were collected within the same measurement context. To test for common method bias, Harman’s-single-factor-test was conducted using PCA (124). The extraction of one factor explains 21.69% of the total variance. As this value is well below the threshold of 50%, no common method bias can be proven (125).

Finally, the statistical analysis needs to be discussed in terms of internal validity. The conditions for imputation were generally favorable and the missing rate was low (1.50%). Thus, no significant bias in imputed values is expected despite the violation of the normal distribution assumption (138). In the presence of ordinal variables, ML estimation tends to produce inflationary χ^2^-values, underestimated factor loadings; correlations; and standard errors, and biased error variances (126, 127). However, the effects are likely to be small due to bootstrapping and the large sample size. In terms of testing for group differences, it should be noted that the measurement models were valid for both groups. Equalization of the sample sizes in gender-stratified groups avoided biased chi-square difference tests.

### 5.4 Strengths and weaknesses

Strengths and weaknesses beyond the biases discussed above are outlined below. With the S-MGA, this study is based on a very well-documented dataset with transregional representation, which allows for comprehensive conclusions across occupations and sectors. As the S-MGA focuses on the relationship between work and mental health, its data were well suited to this study. The outstanding sample size enables precise and robust estimates, even within stratified samples. Moreover, it is essential to highlight the comprehensive approach. Through the multi-faceted analysis of interconnections using SEM, a comprehensive view of the working conditions that arise collectively in practice can be obtained. Including working conditions with cross-functional implications further ensures a high level of generic quality while focusing on issues of particular relevance.

Finally, the inclusion of *quality of leadership* and *subjective perceived stress* should be emphasized at this point. Previous research has mostly focused on social support provided by supervisors. However, the impact of supervisor behavior on employee health extends beyond social support. These determinants are considered in this study. Considering *subjective perceived stress*, it is further possible to identify approaches for counteracting negative demands at an early stage. In contrast to the consideration of long-term consequences of stress, such as burnout, this results in implications that not only address stress but also prevent a variety of stress-related consequences.

Alongside these strengths, there are some limitations to be aware of. First of all, the period of the data collected must be stated. As data were retrieved 2011–2012, the socio-economical context might have changed. Nevertheless, regarding job stressors and resources, studies indicate that gender roles have hardly changed as women (care work, mental load) and men also showed traditional role models in relation to Covid-19 when both worked at home (128, 129). Even though a comprehensive combination of working conditions was included, they still only represent a limited section of workplace reality. Thus, this study provides excellent initial guidance for implementations, but further investigations within the individual professional contexts are recommended. In addition, the cross-sectional design does not allow conclusions to be drawn about causal effects. Longitudinal studies are therefore needed (130, 131). Finally, some limitations regarding the transferability of the results need to be considered.

### 5.5 Transferability

Due to the representative sample, taking into account the limitations outlined above, the findings can be applied to the German labor market almost without restriction. However, other countries differ from German conditions in terms of cultural values and norms, role models, labor law, or even the design of working conditions. Differing gender-specific patterns have been identified in the correlation between work characteristics and mental health across various countries (117). Therefore, the transferability of the results to other countries might be limited.

The restricted age range of the underlying sample, which is between 31 and 60 years, further limits the transferability. The sample does not include young workers, such as apprentices, or workers over the age of 60. However, these target groups are also represented in the labor market. It cannot be excluded that there are other interrelationships for employees beyond the age range considered here. Particularly in view of the increasing importance of apprentices in the next few years (132) and an increase in the retirement age, it is advisable to address these groups in further research.

It should also be noted that the data was collected several years ago. Since then, the generational distribution of employees has changed. As a result, the given stressors and resources are confronted with different role models, ways of thinking, and behaviors. Additionally, working conditions have evolved due to the COVID-19 pandemic and technological advances (133–135). This development impacts the considered stressors and resources. For instance, the requirements for a high *quality of leadership* in times of remote working and the increasing use of artificial intelligence are different from those of a few years ago.

A final point to consider is the combination of stressors and resources. In terms of effect strength relating to gender differences, the results appear to rely on the specific combination of working conditions analyzed. Therefore, this study offers valuable guidance for initial interventions for a wide range of workplaces by considering key conditions of high generic quality. However, varying contexts are found to impact gender differences in stress differently (136). Thus, for a precise comprehension of gender differences in a certain workplace, it may be necessary to conduct a specific analysis that takes into account additional relevant stressors and resources. If there are specific findings available for the target group of interest, these should be prioritized.

## 6 Conclusion

This study sets out to assess gender differences in the effects of job demands and resources on *subjective perceived stress*. Taken together, an increase in *subjective perceived stress* is related to high levels of *quantitative demands* and *working time*, and a decrease in *subjective perceived stress* is associated with an increase in *co-worker support*, *quality of leadership*, and *job control*. This study has demonstrated that these interactions vary between males and females, but the differences are limited. Equivalent for both genders, the most important variables in terms of *subjective perceived stress* are *quantitative demands* and *quality of leadership*. Furthermore, the role of resources can be cautiously assumed to be equal for males and females. The gender gap in the impact of stressors and resources on stress may continue to diminish. Moreover, new evidence is emerging on the stress-reducing effect of a high *quality of leadership* for men and women. Further research is needed to verify the findings. The use of gendered approaches is still strongly recommended, but not necessary in all areas. Gendered approaches are especially recommended in research and in the context of *working time* in organizational practice.

## Data Availability

Publicly available datasets were analyzed in this study. This data can be found at: Federal Institute of Occupational Safety and Health, Germany.
