# Supplementary material for: Gender-specific perception of job stressors and resources: a structural equation model-based secondary analysis
Source: Front Public Health. 2024 Dec 13;12:1463868. doi: 10.3389/fpubh.2024.1463868 (PMC11671497; doi:10.3389/fpubh.2024.1463868)
Supplement: Supplementary file 1 [file Data_Sheet_1.docx]

Supplementary Material

**Standardized Reporting Of Secondary data Analyses (STROSA-2 Checklist)**

|  | **To Find in Line** |
| --- | --- |
| Title, Abstract, Keywords |  |
| 1. Title and Abstract | 55-96 |
| 1. Keywords | 99 |
| Introduction |  |
| 1. Background and Rationale | 109-316 |
| 1. Objective | 140-175 |
| Methods |  |
| 1. Study Design | 322-330 |
| 1. Data Source | 333-368 |
| 1. Legal Basis | 377-378 |
| 1. Data Protection | 379-382 |
| 1. Data Flow | 371-382, |
| 1. Selection Criteria | 385-398 |
| 1. Unit of Analysis | 385-398 |
| 1. Variables | 418-444 |
| 1. Study Size | 466-474 |
| 1. Statistical Methods | 457-504 |
| Results |  |
| 1. Selection of the Study Population | 509-514, 573-611 |
| 1. Descriptive Results | 514-529, 689-724 |
| 1. Main Results | 531-905 |
| 1. Further Results | Not necessarily required, and therefore not included |
| Discussion |  |
| 1. Main results | 910-935 |
| 1. Internal Validity and Risk of Bias | 1099-1160 |
| 1. Strengths and Weaknesses | 1163-1204 |
| 1. Interpretation | 938-1096 |
| 1. Transferability | 1207-1250 |
| Conclusion |  |
| 1. Conclusion | 1252-1274 |
| Conflicts of Interest |  |
| 1. Financing | 1294-1297 |
| 1. Role of Data Owners | 1327-1331 |
| 1. Other Conflicts of Interest | 1334-1338 |
