# Supplementary material for: Gender-specific perception of job stressors and resources: a structural equation model-based secondary analysis
Source: Front Public Health. 2024 Dec 13;12:1463868. doi: 10.3389/fpubh.2024.1463868 (PMC11671497; doi:10.3389/fpubh.2024.1463868)
Supplement: Supplementary file 2 [file Data_Sheet_2.docx]

Supplementary Material

**Supplement A Constructs and Items**

***Supplement A.1 Operationalization of Job Demand Constructs***

| Construct | Item | | Reference |
| --- | --- | --- | --- |
| Quantitative Demands  (QtD) | How often… | | Adapted from the 1st German version of the Copenhagen Psychosocial Questionnaire COPSOQ (Nübling et al., 2005) |
|  | QtD_1 | … do you have enough time for your work tasks?^a^ |  |
|  | QtD_2 | … does your work pile up? |  |
|  | QtD_3 | … do you not have time to complete all your work tasks? |  |
|  | QtD_4 | … do you get behind with your work? |  |
|  | Scale: (1) *always* … (5) *never/ hardly ever* | |  |
| Qualitative Demands  (QlD) | QlD_1 | Coding according to the German Classification of Occupations 2010. | Statistik der Bundesagentur für Arbeit, 2010 |
|  | Scale: (1) *Assistant and semi-skilled jobs*, (2) *Technically oriented jobs*, (3) *Complex specialized jobs*, (4) *Highly complex jobs* | |  |
| Working time  (WT) | WT_1 | How many hours a week do you usually work? |  |
|  | Scale: (1) *less than ten hours*, (2) *short part-time*, (3) *long part-time*, (4) *moderate full-time*, (5) *long full-time*, (6) *excessive full-time up to 60h*, (7) *excessive* *full-time over 60h*^b^ | |  |
|  | | | |
| *Note.* ^a^Opposite coded; ^b^short part-time (10.0-19.9h), long part-time (20.0-34.9h), moderate full-time (35.0-39.9h), long full-time (40.0-48.0), excessive full-time up to 60h (48.1-60.0). | | | |

***Supplement A.2 Operationalization of Job Resources Constructs***

| Construct | Item | | | Reference |
| --- | --- | --- | --- | --- |
| Co-Worker Support  (CS) | How often… | | | 1st German version of the Copenhagen Psychosocial Questionnaire COPSOQ (Nübling et al., 2005) |
|  | CS_1 | | … do you get help and support from your colleagues? |  |
|  | CS_2 | | … are your colleagues willing to listen to your problems at work? |  |
|  | CS_3 | | … do your colleagues talk to you about the quality of your work? |  |
|  | Scale: (1) *never/ hardly ever* … (5) *always* | | |  |
| Quality of Leadership  (QoL) | To what extent does your immediate superior… | | | 1st German version of the Copenhagen Psychosocial Questionnaire COPSOQ (Nübling et al., 2005) |
|  | QoL_1 | | ...make sure that the members of staff have good development opportunities? |  |
|  | QoL_2 | | … gives high priority to job satisfaction? |  |
|  | QoL_3 | | … is good at work planning? |  |
|  | QoL_4 | | … is good at solving conflicts? |  |
|  | Scale: (1) *to a very small extent* … (5) *to a very large extent* | | |  |
| Job Control  (JC) | How often… | | | 1st German version of the Copenhagen Psychosocial Questionnaire COPSOQ (Nübling et al., 2005) |
|  | JC_1 | … do you have any influence on what you do at work? | |  |
|  | JC_2 | … do you have an influence on who you work with? | |  |
|  | JC_3 | … do you have an influence on the amount of work assigned to you? | |  |
|  | Scale: (1) *never/ hardly ever* … (5) *always* | | |  |

***Supplement A.3 Operationalization of Subjective Perceived Stress***

| Construct | Item | | Reference | |
| --- | --- | --- | --- | --- |
| Stress  (SPS) | SPS_1 | Please think about what you have done and experienced in the last four weeks and what you have experienced. Then indicate how often you have experienced each of the following feelings: contented | | Scale of Positive and Negative Experience SPANE (Diener et al., 2010) |
|  | Scale: (1) *very often or always* … (5) *very rarely or never* | | |  |
|  | SPS_2 | I am optimistic about my future | | Psychological Well-Being Scale PWB (Diener et al., 2009) |
|  | Scale: (1*) I strongly agree* … (7) *I strongly disagree* | | |  |
|  | SPS_3 | Think about the last four weeks. During this time, how often have you felt a lot of energy? | | Short-Form Health Survey SF-12 (Nübling et al.,  2006; Schupp, 2005) |
|  | Scale: (1) *always* … (5) *never* | | |  |
|  | SPS_4 | Think about the last four weeks. During this time, how often have you felt calm and peaceful? | | Short-Form Health Survey SF-12 (Nübling et al.,  2006; Schupp, 2005) |
|  | Scale: (1) *always* … (5) *never* | | |  |

References to the items used

Diener, E., Wirtz, D., Biswas-Diener, R., Tov, W., Kim-Prieto, C., Choi, D., & Oishi, S. (2009). New Measures of Well-Being. In E. Diener (Ed.), Assessing Well-Being (Vol. 39, pp. 247–266). Springer Netherlands. https://doi.org/10.1007/978-90-481-2354-4_12

Diener, E., Wirtz, D., Tov, W., Kim-Prieto, C., Choi, D., Oishi, S., & Biswas-Diener, R. (2010). New Well-being Measures: Short Scales to Assess Flourishing and Positive and Negative Feelings. Social Indicators Research, 97(2), 143–156. https://doi.org/10.1007/s11205-009-9493-y

Nübling, M., Andersen, H. H., & Mühlbacher, A. (2006). Entwicklung eines Verfahrens zur Berechnung der körperlichen und psychischen Summenskalen auf Basis der SOEP – Version des SF 12 (Algorithmus). DIW Data Documentation, No. 16. Berlin: Deutsches Institut für Wirtschaftsforschung (DIW).

Nübling, M., Stößel, U., Hasselhorn, H.-M., Michaelis, M., & Hofmann, F. (2005). Methoden zur Erfassung psychischer Belastungen: Erprobung eines Messinstrumentes (COPSOQ): Erprobung eines Messinstrumentes (COPSOQ) (Vol. 1058). Wirtschaftsverl. NW Verl. für Neue Wiss.

Schupp, J. (2005). Befragungsgestützte Messung von Gesundheit – Bestandsausnahme und Ausblick. Workshop am DIW Berlin am 14. März 2005. DIW Eventdokumentation, No. 2. Berlin: Deutsches Institut für Wirtschaftsforschung (DIW).

Statistik der Bundesagentur für Arbeit (Ed.). (2010). *German Classification of Occupations 2010*.

***Supplement B Covariates***

| Covariates | Categories/Groups |
| --- | --- |
| Age (continuous) | *31-35 years, 36-40 years, 41-45 years, 46-50 years, 51-55 years, 56-60 years* |
| Educational Level (categorial) | *no education* = 1*, completed vocational training in a company (apprenticeship) = 2, completed vocational training in a school (vocational or commercial school) = 3, graduated from a specialised, master or technical school, vocational or technical college = 4, graduated from a university of applied sciences = 5,* *university degree* = 6 |
| Income (categorial) | *up to 400 euros* = 1*, over 400 euros to 800 euros = 2, over 800 euros to under 1,000 euros = 3, 1,000 euros to under 1,500 euros = 4, 1,500 euros to under 2,000 euros = 5, 2,000 euros to under 2,500 euros = 6, 2,500 euros to under 3,000 = 7, 3,000 euros to under 4,000 euros = 8, 4,000 euros to under 5,000 euros = 9,* *5,000 euros and more* = 10 |
| Children under 14 in the household (categorial) | *none* = 0, *one* = 1, *two* = 2, *three or more* = 3 |
| Caregiving (categorial) | *yes* = 1, *no* = 2 |
| Hours worked in paid second jobs (continuous) | *less than ten hours = 1*, *short part-time = 2*, *long part-time = 3*, *moderate full-time = 4*, *long full-time = 5*, *excessive full-time up to 60h = 6*, *excessive* *full-time over 60h = 7* |

**Supplement C Missing Values**

***C.1 Descriptive Analysis of Indicators Before EM Imputation***

| Construct | Items | Missing values | | Averages (SD)^a^ | | Skewness (C.R.) | | Kurtosis (C.R.) |
| --- | --- | --- | --- | --- | --- | --- | --- | --- |
| QtD | QtD_1 | 10 | | 2.67 (1.15) | | 0.27 (6.99) | | -0.84 (-11.05) |
|  | QtD_2 | 10 | | 3.00 (1.10) | | -0.22 (-5.81) | | -0.65 (-8.49) |
|  | QtD_3 | 9 | | 2.93 (1.15) | | -0.09 (-2.27) | | -0.83 (-10.84) |
|  | QtD_4 | 7 | | 2.33 (1.09) | | 0.48 (12.65) | | -0.51 (-6.70) |
| QlD | QlD_1 | 14 | | 2.00 (1-4) | | 0.54 (14.22) | | -0.47 (-6.15) |
| WT | WT_1 | 16 | | 5.00 (1-7) | | -0.44 (-11.44) | | -0.65 (-8.56) |
| QoL | QoL_1 | 61 | | 3.23 (1.16) | | -0.29 (-7.60) | | -0.72 (-9.32) |
|  | QoL_2 | 62 | | 3.48 (1.09) | | -0.57 (-14.79) | | -0.27 (-3.53) |
|  | QoL_3 | 168 | | 3.25 (1.12) | | -0.40 (-10.33) | | -0.53 (-6.77) |
|  | QoL_4 | 137 | | 3.21 (1.13) | | -0.32 (-8.23) | | -0.61 (-7.89) |
| CS | CS_1 | 1 | | 3.71 (1.19) | | -0.75 (-19.59) | | -0.28 (-3.71) |
|  | CS_2 | 18 | | 3.69 (1.21) | | -0.77 (-20.09) | | -0.30 (-3.86) |
|  | CS_3 | 11 | | 2.93 (1.21) | | -0.03 (-0.90) | | -0.96 (-12.50) |
| JC | JC_1 | 13 | | 2.95 (1.44) | | -0.06 (-1.55) | | -1.37 (-17.93) |
|  | JC_2 | 19 | | 2.12 (1.34) | | 0.89 (23.32) | | -0.54 (-7.05) |
|  | JC_3 | 7 | | 2.08 (1.22) | | 0.88 (23.03) | | -0.35 (-4.57) |
| SPS | SPS_1 | 405 | | 2.32 (0.82) | | 0.74 (18.42) | | 0.76 (9.51) |
|  | SPS_2 | 400 | | 2.59 (1.25) | | 1.12 (27.84) | | 1.26 (15.71) |
|  | SPS_3 | 6 | | 2.73 (0.81) | | 0.23 (6.11) | | -0.05 (-0.71) |
|  | SPS_4 | 3 | | 2.59 (0.85) | | 0.40 (10.45) | | -0.16 (-2.15) |
| grouping variable | gender | 0 | | 1.50 (0.50) | | 0.00 (0.00) | | -2.00 (-26.23) |
| Confounder | year of birth | 0 | | 1963 (1951-1980) | | 0.14 (3.79) | | -1.00 (-13.08) |
|  | educational level | 1 | | 2.00 (1-6) | | 0.79 (20.63) | | -0.72 (-9.49) |
|  | income | 279 | | 5.00 (1-10) | | 0.17 (4.30) | | -0.08 (-0.97) |
|  | children < 14 | 4 | | 0.00 (0-3) | | 1.53 (40.21) | | 1.30 (17.09) |
|  | caregiving | 0 | | 2.00 (1-2) | | -6.22 (-162.93) | | 36.67 (480.59) |
|  | working hours second jobs | 2 | | 0.00 (1-7) | | 7.43 (194.78) | | 63.50 (832.03) |
|  |  | |  |  |  | |  | |
| *Note.* *n* = 4,118. QtD = quantitative demands, QlD = qualitative demands, WT = working time; JC = job control, QoL = quality of leadership, CS = co-worker support, SPS = subjective perceived stress, C.R. = Critical Ratio; ^a^Means indicate the average for metric items, median was calculated for ordinal items. SD values were computed for metric items only. Range was calculated for ordinal items as measure of dispersion. | | | | | | | | |

***C.2 Descriptive Analysis of Indicators After EM Imputation***

| Construct | Items | | Averages (SD)^a^ | | Skewness (C.R.) | | Kurtosis (C.R.) |
| --- | --- | --- | --- | --- | --- | --- | --- |
| QtD | QtD_1 | | 2.67 (1.15) | | 0.27 (6.98) | | -0.84 (-11.04) |
|  | QtD_2 | | 3.00 (1.10) | | -0.22 (-5.77) | | -0.65 (-8.49) |
|  | QtD_3 | | 2.93 (1.15) | | -0.09 (-2.25) | | -0.82 (-10.80) |
|  | QtD_4 | | 2.33 (1.08) | | 0.48 (12.70) | | -0.51 (-6.67) |
| QlD | QlD_1 | | 2.00 (1-4) | | 0.54 (14.21) | | -0.47 (-6.12) |
| WT | WT_1 | | 5.00 (1-7) | | -.43 (-11.39) | | -.65 (-8.57) |
| QoL | QoL_1 | | 3.23 (1.15) | | -0.29 (-7.69) | | -0.70 (-9.18) |
|  | QoL_2 | | 3.48 (1.08) | | -0.57 (-14.82) | | -0.25 (-3.33) |
|  | QoL_3 | | 3.25 (1.11) | | -0.41 (-10.74) | | -0.47 (-6.22) |
|  | QoL_4 | | 3.21 (1.12) | | -0.32 (-8.48) | | -0.57 (-7.51) |
| CS | CS_1 | | 3.71 (1.19) | | -0.75 (-19.60) | | -0.28 (-3.71) |
|  | CS_2 | | 3.69 (1.21) | | -0.77 (-20.08) | | -0.29 (-3.86) |
|  | CS_3 | | 2.93 (1.21) | | -0.03 (-0.90) | | -0.95 (-12.47) |
| JC | JC_1 | | 2.95 (1.44) | | -0.06 (-1.52) | | -1.37 (-17.92) |
|  | JC_2 | | 2.12 (1.34) | | 0.89 (23.34) | | -0.54 (-7.02) |
|  | JC_3 | | 2.08 (1.22) | | 0.88 (23.00) | | -0.35 (-4.61) |
| SPS | SPS_1 | | 2.32 (0.79) | | 0.75 (19.78) | | 0.97(12.72) |
|  | SPS_2 | | 2.60 (1.21) | | 1.11 (28.99) | | 1.37 (17.93) |
|  | SPS_3 | | 2.73 (0.81) | | 0.23 (6.13) | | -0.05 (-0.71) |
|  | SPS_4 | | 2.59 (0.85) | | 0.40 (10.45) | | -0.16 (-2.15) |
| grouping variable | gender | | 1.50 (1-2) | | 0.00 (0.00) | | -2.00 (-26.33) |
| Confounder | year of birth | | 1963 (1951-1980) | | 0.14 (3.79) | | -1.00 (-13.08) |
|  | educational level | | 2.00 (1-6) | | 0.79 (20.64) | | -0.72 (-9.48) |
|  | income | | 5.00 (1-10) | | 0.15 (3.85) | | -0.07 (-0.89) |
|  | children < 14 | | 0.00 (0-3) | | 1.54 (40.24) | | 1.31 (17.14) |
|  | caregiving | | 2.00 (1-2) | | -6.22 (-162.93) | | 36.67 (480.59) |
|  | working hours second jobs | | 0.00 (1-7) | | 7.43 (185.75) | | 63.43 (792.88) |
|  |  |  | |  | |  | |
| *Note.* *n* = 4,118. QtD = quantitative demands QlD = qualitative demands, WT = working time; JC = job control, QoL = quality of leadership, CS = co-worker support, SPS = subjective perceived stress C.R. = Critical Ratio; ^a^Means indicate the average for metric items, median was calculated for ordinal items. SD values were computed for metric items only. Range was calculated for ordinal items as measure of dispersion. | | | | | | | |

***C.3 Little's MCAR Test***

χ^2^: 2975.85; df = 2657; Sig.: *p* < .001.

**Supplement D Evaluation of Measurement Models**

**D.1 Principal component analysis**

***D.1.1 Total Sample***

Kaiser–Meyer–Olkin value: .82; Bartlett-Test: *p* < .001

| Construct | Item | | MSA | | Communality | Indicator loadings | | | | | | | | | |
| --- | --- | --- | --- | --- | --- | --- | --- | --- | --- | --- | --- | --- | --- | --- | --- |
|  |  | |  | |  | 1 | | 2 | | 3 | | 4 | | 5 | |
| QtD | | QtD_1 | .87 | .64 | | **.78** | | -.01 | | .03 | | .00 | | -.06 | |
|  |  | QtD_2 | .89 | .59 | | **.76** | | -.05 | | -.02 | | .00 | | .01 | |
|  |  | QtD_3 | .80 | .76 | | **.88** | | .02 | | -.01 | | .00 | | .00 | |
|  |  | QtD_4 | .83 | .70 | | **.85** | | .01 | | -.02 | | .00 | | .03 | |
| CS | | CS_1 | .74 | .71 | | .04 | | .02 | | .00 | | -.03 | | **.77** | |
|  |  | CS_2 | .70 | .75 | | .04 | | -.03 | | -.01 | | .02 | | **.78** | |
|  |  | CS_3 | .82 | .57 | | -.10 | | .00 | | .02 | | .01 | | **.80** | |
| QoL | | QoL_1 | .86 | .71 | | .06 | | **.82** | | -.02 | | .05 | | .06 | |
|  |  | QoL_2 | .86 | .72 | | .01 | | **.83** | | -.03 | | .00 | | .04 | |
|  |  | QoL_3 | .88 | .66 | | -.10 | | **.81** | | .04 | | -.02 | | -.08 | |
|  |  | QoL_4 | .87 | .69 | | .01 | | **.84** | | .00 | | -.02 | | -.02 | |
| JC | | JC_1 | .75 | .59 | | -.02 | | .03 | | -.01 | | **.83** | | .00 | |
|  |  | JC_2 | .73 | .61 | | -.01 | | -.02 | | -.02 | | **.87** | | -.03 | |
|  |  | JC_3 | .74 | .65 | | .03 | | .00 | | .04 | | **.75** | | .03 | |
| SPS | | SPS_1 | .85 | .59 | | -.04 | | -.06 | | **.76** | | -.01 | | .03 | |
|  |  | SPS_2 | .85 | .50 | | -.10 | | -.02 | | **.71** | | -.02 | | -.03 | |
|  |  | SPS_3 | .82 | .60 | | -.01 | | .01 | | **.78** | | .03 | | -.02 | |
|  |  | SPS_4 | .82 | .62 | | .15 | | .07 | | **.76** | | .00 | | .03 | |
| Factor eigenvalue (before rotating) | | | | | | | 4.33 | | 2.45 | | 1.78 | | 1.56 | | 1.52 |
| Sum of squared loads (after rotating) | | | | | | | 3.05 | | 3.34 | | 2.83 | | 2.37 | | 2.07 |
| Explained variance | | | | | | | 64.67% | | | | | | | | |
|  | | | | | | | | | | | | | | | |
| *Note.* *n* = 4,118. Extraction method: principal component analysis, rotation method: Promax with Kaiser Normalization. Factors were extracted using the Kaiser criterion. Factor loadings above .50 are in bold. QtD = quantitative demands, CS = co-worker support, QoL = quality of leadership, JC = job control, SPS = subjective perceived stress, MSA = measure of sampling adequacy. | | | | | | | | | | | | | | | |

**D1.2 *Male Sample***

Kaiser–Meyer–Olkin value: .83; Bartlett-Test: *p* < .001

| Construct | Item | | MSA | | Communality | | Indicator loadings | | | | | | | | | |  |
| --- | --- | --- | --- | --- | --- | --- | --- | --- | --- | --- | --- | --- | --- | --- | --- | --- | --- |
|  |  | |  | |  | | 1 | | 2 | | 3 | | 4 | | 5 | |  |
| QtD | | QtD_1 | .88 | .65 | | | **.78** | | -.03 | | .03 | | .01 | | -.06 | |  |
|  |  | QtD_2 | .90 | .58 | | | **.75** | | -.04 | | -.01 | | .00 | | .02 | |  |
|  |  | QtD_3 | .80 | .76 | | | **.88** | | .02 | | -.02 | | .01 | | -.01 | |  |
|  |  | QtD_4 | .83 | .70 | | | **.85** | | .01 | | -.02 | | -.01 | | .02 | |  |
| CS | | CS_1 | .77 | .66 | | | -.03 | | .07 | | .02 | | **.79** | | .02 | |  |
|  |  | CS_2 | .72 | .71 | | | -.01 | | -.04 | | -.02 | | **.85** | | -.01 | |  |
|  |  | CS_3 | .80 | .57 | | | .04 | | -.02 | | .01 | | **.77** | | -.01 | |  |
| QoL | | QoL_1 | .85 | .68 | | | .06 | | **.81** | | -.02 | | .02 | | .06 | |  |
|  |  | QoL_2 | .85 | .70 | | | .01 | | **.82** | | -.01 | | .01 | | .07 | |  |
|  |  | QoL_3 | .87 | .64 | | | -.12 | | **.79** | | .05 | | -.02 | | -.10 | |  |
|  |  | QoL_4 | .88 | .65 | | | .02 | | **.80** | | -.03 | | .00 | | -.04 | |  |
| JC | | JC_1 | .73 | .61 | | | .04 | | .00 | | .01 | | -.03 | | **.78** | |  |
|  |  | JC_2 | .75 | .59 | | | .05 | | .00 | | -.03 | | .04 | | **.76** | |  |
|  |  | JC_3 | .74 | .64 | | | -.11 | | -.01 | | .02 | | -.01 | | **.80** | |  |
| SPS | | SPS_1 | .85 | .59 | | | -.06 | | -.07 | | **.76** | | .02 | | .03 | |  |
|  |  | SPS_2 | .85 | .50 | | | -.11 | | -.02 | | **.71** | | .01 | | -.06 | |  |
|  |  | SPS_3 | .82 | .59 | | | -.01 | | -.01 | | **.77** | | .00 | | .00 | |  |
|  |  | SPS_4 | .82 | .64 | | | .20 | | .09 | | **.75** | | -.02 | | .03 | |  |
| Factor eigenvalue (before rotating) | | | | | | | | 4.36 | | 2.40 | | 1.71 | | 1.51 | | 1.49 | |
| Sum of squared loads (after rotating) | | | | | | | | 3.14 | | 3.27 | | 2.86 | | 2.32 | | 2.07 | |
| Explained variance | | | | | | 63.66% | | | | | | | | | | | |
|  | | | | | | | | | | | | | | | | | |
| *Note.* *n* = 2,059. Extraction method: principal component analysis, rotation method: Promax with Kaiser Normalization. Factors were extracted using the Kaiser criterion. Factor loadings above .50 are in bold. QtD = quantitative demands, CS = co-worker support, QoL = quality of leadership, JC = job control, SPS = subjective perceived stress, MSA = measure of sampling adequacy. | | | | | | | | | | | | | | | | | |

***D1.3 Female Sample***

Kaiser–Meyer–Olkin value: .82; Bartlett-Test: *p* < .001

| Construct | Item | | MSA | | Communality | Indicator loadings | | | | | | | | | |  |
| --- | --- | --- | --- | --- | --- | --- | --- | --- | --- | --- | --- | --- | --- | --- | --- | --- |
|  |  | |  | |  | 1 | | 2 | | 3 | | 4 | | 5 | |  |
| QtD | QtD_1 | .86 | | .63 | | .00 | | **.78** | | .03 | | .00 | | -.04 | |  |
|  | QtD_2 | .88 | | .59 | | -.06 | | **.76** | | -.02 | | .00 | | -.02 | |  |
|  | QtD_3 | .79 | | .76 | | .02 | | **.88** | | .00 | | -.01 | | .01 | |  |
|  | QtD_4 | .82 | | .70 | | .02 | | **.85** | | -.02 | | .00 | | .04 | |  |
| CS | CS_1 | .71 | | .75 | | .03 | | .04 | | -.02 | | -.03 | | **.76** | |  |
|  | CS_2 | .69 | | .78 | | -.04 | | .03 | | .00 | | .01 | | **.79** | |  |
|  | CS_3 | .83 | | .57 | | .00 | | -.10 | | .01 | | .02 | | **.80** | |  |
| QoL | QoL_1 | .87 | | .73 | | **.82** | | .05 | | -.01 | | .07 | | .06 | |  |
|  | QoL_2 | .86 | | .74 | | **.84** | | .00 | | -.05 | | -.01 | | .02 | |  |
|  | QoL_3 | .89 | | .69 | | **.84** | | -.07 | | .04 | | -.02 | | -.06 | |  |
|  | QoL_4 | .86 | | .74 | | **.88** | | .00 | | .02 | | -.03 | | -.03 | |  |
| JC | JC_1 | .75 | | .58 | | .00 | | -.02 | | -.03 | | **.86** | | -.02 | |  |
|  | JC_2 | .71 | | .61 | | -.02 | | -.01 | | -.02 | | **.89** | | -.04 | |  |
|  | JC_3 | .74 | | .65 | | .03 | | .03 | | .07 | | **.74** | | .07 | |  |
| SPS | SPS_1 | .84 | | .61 | | -.04 | | -.01 | | **.77** | | -.04 | | .02 | |  |
|  | SPS_2 | .84 | | .51 | | -.01 | | -.09 | | **.72** | | -.04 | | -.02 | |  |
|  | SPS_3 | .81 | | .60 | | .02 | | -.01 | | **.78** | | .06 | | -.04 | |  |
|  | SPS_4 | .82 | | .62 | | .03 | | .12 | | **.77** | | .03 | | .04 | |  |
| Factor eigenvalue (before rotating) | | | | | | | 4.32 | | 2.55 | | 1.81 | | 1.63 | | 1.53 | |
| Sum of squared loads (after rotating) | | | | | | | 3.43 | | 2.98 | | 2.83 | | 2.45 | | 2.07 | |
| Explained variance | | | | | | | 65.76% | | | | | | | | |  |
|  | | | | | | | | | | | | | | | | |
| *Note. n* = 2,059. Extraction method: principal component analysis, rotation method: Promax with Kaiser Normalization. Factors were extracted using the Kaiser criterion. Factor loadings above .50 are in bold. QtD = quantitative demands, CS = co-worker support, QoL = quality of leadership, JC = job control, SPS = subjective perceived stress, MSA = measure of sampling adequacy. | | | | | | | | | | | | | | | | |

***D.2* C*onfirmatory Factor Analysis***

***D.2.1 Model of Confirmatory Factor Analysis***


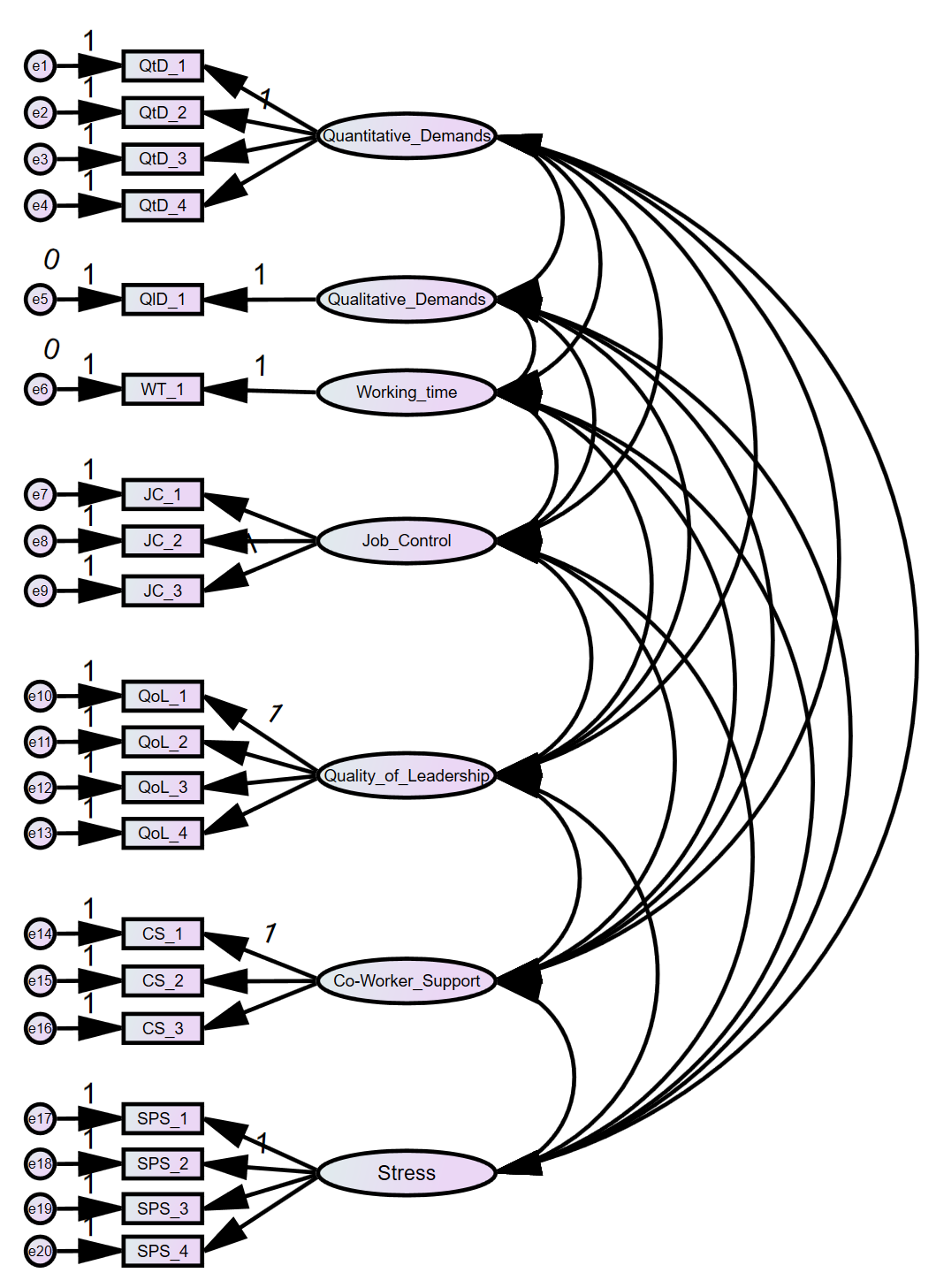


*Note.* QtD = quantitative demands, QlD = qualitative demands, WT = working time, CS = co-worker support, QoL = quality of leadership, JC = job control, SPS = subjective perceived stress. Adjusted for age, educational level, income, children under 14 years in the household, caregiving, hours worked in paid second jobs.

***D.2.2 Total* Sample**

| Construct | Item | CFA results | | Reliability calculations | | |
| --- | --- | --- | --- | --- | --- | --- |
|  |  | Standardized factor loading | Error variance | SMC | CR | AVE |
| QtD  (Variance: 1,0) | QtD_1 | .71 | .50 | .50 | .84 | .57 |
|  | QtD_2 | .67 | .55 | .45 |  |  |
|  | QtD_3 | .84 | .29 | .71 |  |  |
|  | QtD_4 | .78 | .39 | .61 |  |  |
| CS  (Variance: 1,0) | CS_1 | .77 | .41 | .59 | .76 | .52 |
|  | CS_2 | .81 | .34 | .66 |  |  |
|  | CS_3 | .57 | .68 | .32 |  |  |
| QoL  (Variance: 1,0) | QoL_1 | .78 | .39 | .61 | .85 | .58 |
|  | QoL_2 | .80 | .36 | .64 |  |  |
|  | QoL_3 | .71 | .50 | .50 |  |  |
|  | QoL_4 | .75 | .44 | .56 |  |  |
| JC  (Variance: 1,0) | JC_1 | .63 | .60 | .40 | .68 | .42 |
|  | JC_2 | .63 | .60 | .40 |  |  |
|  | JC_3 | .68 | .54 | .46 |  |  |
| SPS  (Variance: 1,0) | SPS_1 | .66 | .56 | .44 | .75 | .43 |
|  | SPS_2 | .56 | .69 | .31 |  |  |
|  | SPS_3 | .68 | .54 | .36 |  |  |
|  | SPS_4 | .71 | .50 | .50 |  |  |
|  | | | | | | |
| *Note. n* = 4,118. QtD = quantitative demands, CS = co-worker support, QoL = quality of leadership, JC = job control, SPS = subjective perceived stress, SMC = squared multiple correlation, CR = composite reliability, AVE = Average Variance Extracted; Estimation method: ML; **Model Fit Statistics:** χ^2^ = 1437.76, df = 237, CFI = .96, RMSEA = .04, SRMR = .03, AGFI = .96. | | | | | | |

***D.2.3 Male Sample***

| Construct | Item | CFA results | | | Reliability calculations | | |
| --- | --- | --- | --- | --- | --- | --- | --- |
|  |  | Standardized factor loading | Error variance | SMC | | CR | AVE |
| QtD  (Variance: 1,0) | QtD_1 | .72 | .48 | .52 | | .84 | .57 |
|  | QtD_2 | .66 | .56 | .44 | |  |  |
|  | QtD_3 | .84 | .29 | .71 | |  |  |
|  | QtD_4 | .79 | .38 | .62 | |  |  |
| CS  (Variance: 1,0) | CS_1 | .72 | .48 | .52 | | .73 | .47 |
|  | CS_2 | .77 | .41 | .59 | |  |  |
|  | CS_3 | .56 | .69 | .31 | |  |  |
| QoL  (Variance: 1,0) | QoL_1 | .76 | .42 | .58 | | .83 | .55 |
|  | QoL_2 | .79 | .38 | .62 | |  |  |
|  | QoL_3 | .68 | .54 | .46 | |  |  |
|  | QoL_4 | .72 | .48 | .52 | |  |  |
| JC  (Variance: 1,0) | JC_1 | .64 | .59 | .41 | | .68 | .41 |
|  | JC_2 | .62 | .62 | .38 | |  |  |
|  | JC_3 | .67 | .55 | .45 | |  |  |
| SPS  (Variance: 1,0) | SPS_1 | .64 | .59 | .41 | | .74 | .42 |
|  | SPS_2 | .55 | .70 | .30 | |  |  |
|  | SPS_3 | .68 | .54 | .46 | |  |  |
|  | SPS_4 | .72 | .48 | .52 | |  |  |
|  |  |  |  |  | |  |  |
| *Note. n* = 2,059. QtD = quantitative demands, CS = co-worker support, QoL = quality of leadership, JC = job control, SPS = subjective perceived stress, SMC = squared multiple correlation, CR = composite reliability, AVE = Average Variance Extracted; Estimation method: ML; **Model Fit Statistics:** χ^2^ = 891.38, df = 237, CFI = .96, RMSEA = .04, SRMR = 04, AGFI = .95. | | | | | | | |

**D.2.4 Female Sample**

| Construct | Item | CFA results | | | Reliability calculations | | |
| --- | --- | --- | --- | --- | --- | --- | --- |
|  |  | Standardized factor loading | Error variance | SMC | | CR | AVE |
| QtD  (Variance: 1,0) | QtD_1 | .70 | .51 | .49 | | .84 | .57 |
|  | QtD_2 | .68 | .54 | .46 | |  |  |
|  | QtD_3 | .84 | .29 | .71 | |  |  |
|  | QtD_4 | .78 | .39 | .61 | |  |  |
| CS  (Variance: 1,0) | CS_1 | .80 | .36 | .64 | | .80 | .57 |
|  | CS_2 | .86 | .26 | .74 | |  |  |
|  | CS_3 | .58 | .66 | .34 | |  |  |
| QoL  (Variance: 1,0) | QoL_1 | .80 | .36 | .64 | | .87 | .62 |
|  | QoL_2 | .82 | .33 | .67 | |  |  |
|  | QoL_3 | .74 | .45 | .55 | |  |  |
|  | QoL_4 | .79 | .38 | .62 | |  |  |
| JC  (Variance: 1,0) | JC_1 | .61 | .63 | .37 | | .68 | .42 |
|  | JC_2 | .61 | .63 | .37 | |  |  |
|  | JC_3 | .71 | .50 | .50 | |  |  |
| SPS  (Variance: 1,0) | SPS_1 | .69 | .52 | .48 | | .76 | .44 |
|  | SPS_2 | .58 | .66 | .34 | |  |  |
|  | SPS_3 | .67 | .55 | .45 | |  |  |
|  | SPS_4 | .70 | .51 | .49 | |  |  |
|  |  |  |  |  | |  |  |
| *Note. n* = 2,059. QtD = quantitative demands, CS = co-worker support, QoL = quality of leadership, JC = job control, SPS = subjective perceived stress, SMC = squared multiple correlation, CR = composite reliability, AVE = Average Variance Extracted; Estimation method: ML; **Model Fit Statistics:** χ^2^ = 810.27, df = 237, CFI = .97, RMSEA = .03, SRMR = .03, AGFI = .95. | | | | | | | |

***D.3 Heterotrait-Monotrait Ratio of Correlations***

**D.3.1 Total Sample**

| Construct | QtD | QlD | WT | CS | QoL | JC | SPS |
| --- | --- | --- | --- | --- | --- | --- | --- |
| QtD | – |  |  |  |  |  |  |
| QlD | .27 | – |  |  |  |  |  |
| WT | .23 | .31 | – |  |  |  |  |
| CS | -.05 | .10 | .07 | – |  |  |  |
| QoL | -.29 | -.01 | -.07 | .26 | – |  |  |
| JC | -.05 | .36 | .16 | .23 | .22 | – |  |
| SPS | .33 | -.09 | .01 | -.23 | -.39 | -.26 | – |
|  |  |  |  |  |  |  |  |
| *Note.* *n* = 4,118. QtD = quantitative demands QlD = qualitative demands, WT = working time; JC = job control, QoL = quality of leadership, CS = co-worker support, SPS = subjective perceived stress. | | | | | | | |

**D.3.2 Male Sample**

| Construct | QtD | QlD | WT | CS | QoL | JC | SPS |
| --- | --- | --- | --- | --- | --- | --- | --- |
| QtD | – |  |  |  |  |  |  |
| QlD | .30 | – |  |  |  |  |  |
| WT | .23 | .27 | – |  |  |  |  |
| CS | -.12 | .04 | .02 | – |  |  |  |
| QoL | -.32 | -.01 | -.03 | .38 | – |  |  |
| JC | -.00 | .39 | .13 | .24 | .24 | – |  |
| SPS | .36 | -.09 | -.02 | -.27 | -.40 | -.26 | – |
|  |  |  |  |  |  |  |  |
| *Note. n* = 2,059. QtD = quantitative demands QlD = qualitative demands, WT = working time; JC = job control, QoL = quality of leadership, CS = co-worker support, SPS = subjective perceived stress. | | | | | | | |

**D.3.3 Female Sample**

| Construct | QtD | QlD | WT | CS | QoL | JC | SPS |
| --- | --- | --- | --- | --- | --- | --- | --- |
| QtD | – |  |  |  |  |  |  |
| QlD | .24 | – |  |  |  |  |  |
| WT | .26 | .30 | – |  |  |  |  |
| CS | .01 | .14 | .08 | – |  |  |  |
| QoL | -.26 | .00 | -.07 | .35 | – |  |  |
| JC | -.11 | .31 | .11 | .21 | .22 | – |  |
| SPS | .30 | -.08 | .06 | -.19 | -.37 | -.25 | – |
|  |  |  |  |  |  |  |  |
| *Note. n* = 2,059. QtD = quantitative demands QlD = qualitative demands, WT = working time; JC = job control, QoL = quality of leadership, CS = co-worker support, SPS = subjective perceived stress. | | | | | | | |

**Supplement E Tests of Measurement Invariance Across Gender**

| Model | χ^2^ (df) | | CFI | | RMSEA | | SRMR | | Reference model | | Δχ^2^ (Δdf) | | | Δ CFI | | Δ RMSEA | | Δ SRMR | Decision | |  |
| --- | --- | --- | --- | --- | --- | --- | --- | --- | --- | --- | --- | --- | --- | --- | --- | --- | --- | --- | --- | --- | --- |
| M^U^: Configural Invariance | 1704.79 (475) | | .96 | | .03 | | .04 | | – | | – | | | – | | – | | – | accepted | |  |
| M^M^: Metric Invariance | 1772.15 (493) | | .96 | | .03 | | .04 | | M^U^ | | 67.37^***^ (18) | | | .00 | | .00 | | .00 | accepted | |  |
|  | |  | |  | |  | |  | |  | |  |  | |  | |  | | |  | |
| *Note. n* = 4,118. M^U^ = Unconstrained Modell, M^M^ = Measurement Weights Modell, χ^2^ = = chi-square value, CFI = Comparative Fit Index, RMSEA = Root Mean Square Error of Approximation, SRMR = Standardized Root Mean Squared Residual; Estimation method: ML; ****p* < .001. | | | | | | | | | | | | | | | | | | | |  |  |

**Supplement F Structural Equation Model**

**
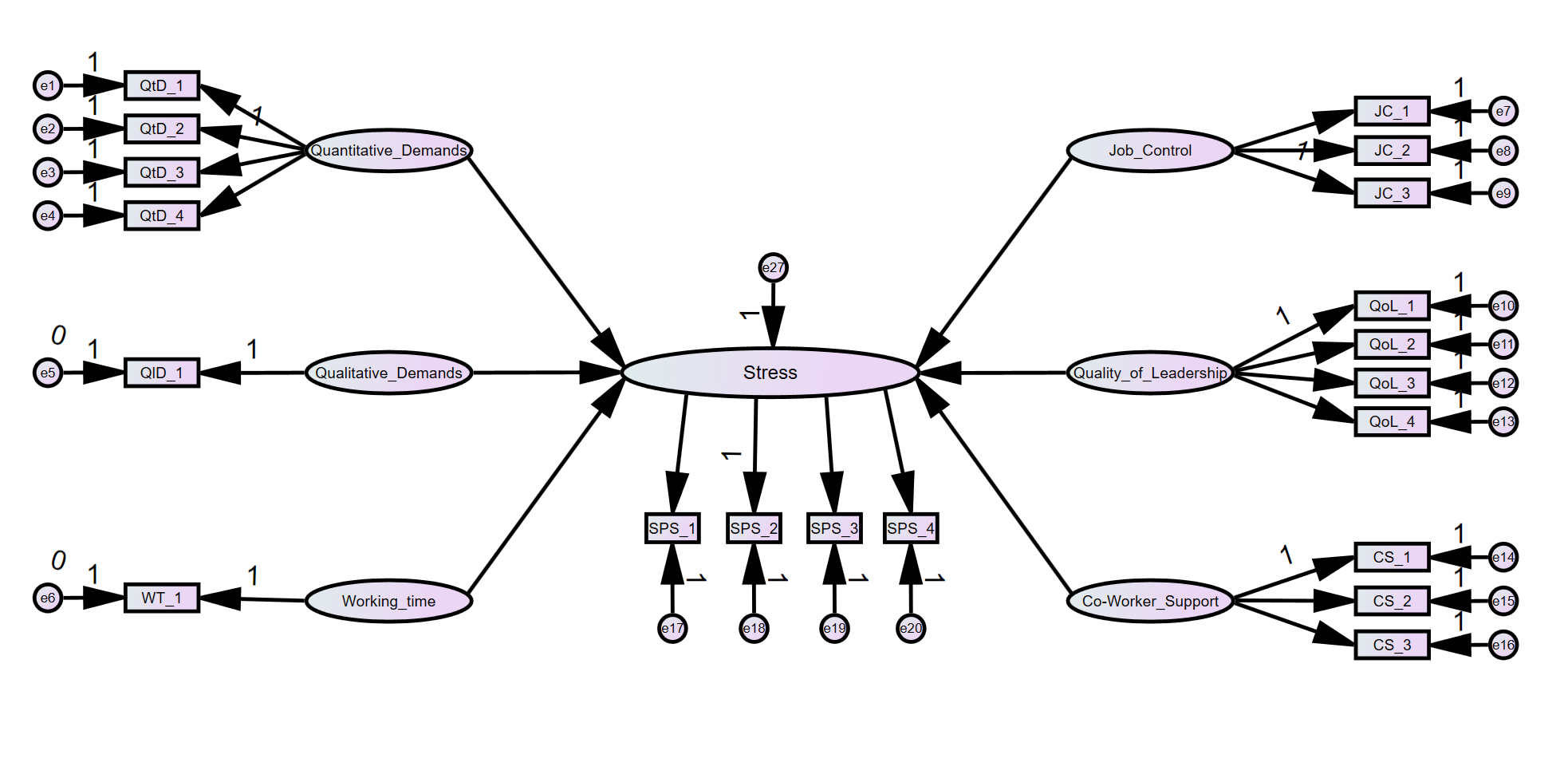
**

*Note.* QtD = quantitative demands, QlD = qualitative demands, WT = working time, CS = co-worker support, QoL = quality of leadership, JC = job control, SPS = subjective perceived stress. Adjusted for age, educational level, income, children under 14 years in the household, caregiving, hours worked in paid second jobs.

**Supplement G Tests of Measurement Invariance**

| Model | | χ^2^  (df) | CFI | | RMSEA | | SRMR | | Reference model | | Δχ^2^  (Δdf) | | ΔCFI | | ΔRMSEA | | ΔSRMR | | |  |
| --- | --- | --- | --- | --- | --- | --- | --- | --- | --- | --- | --- | --- | --- | --- | --- | --- | --- | --- | --- | --- |
| M^U^:  Baseline Model | | 3648.82 (498) | .90 | | .04 | | .0898 | | – | | – | | – | | – | | – | | |  |
| M1: Structural Invariance | | 3683.03 (517) | .90 | | .04 | | .0903 | | M^U^ | | 34.21^*^ (19) | | .00 | | .00 | | .00 | | |  |
| M2:  Path QtD🡪SPS constrained | | 3654.75 (499) | .90 | | .04 | | .0898 | | M^U^ | | 5.93^*^  (1) | | .00 | | .00 | | .00 | | |  |
| M3:  Path QlD🡪SPS constrained | | 3649.47 (499) | .90 | | .04 | | .0898 | | M^U^ | | 0.64  (1) | | .00 | | .00 | | .00 | | |  |
| M4:  Path WT🡪SPS constrained | | 3652.38 (499) | .90 | | .04 | | .0898 | | M^U^ | | 3.55  (1) | | .00 | | .00 | | .00 | | |  |
| M5:  Path CS🡪SPS constrained | | 3651.81 (499) | .90 | | .04 | | .0900 | | M^U^ | | 2.99  (1) | | .00 | | .00 | | .00 | | |  |
| M6:  Path QoL🡪SPS constrained | | 3648.84 (499) | .90 | | .04 | | .0898 | | M^U^ | | 0.02  (1) | | .00 | | .00 | | .00 | | |  |
| M7:  Path JC🡪SPS constrained | | 3648.85 (499) | .90 | | .04 | | .0897 | | M^U^ | | 0.02  (1) | | .00 | | .00 | | .00 | | |  |
|  |  | | |  | |  | |  | |  | |  | |  | |  | |  | | |
| *Note. n* = 4,118. QtD = quantitative demands, QlD = qualitative demands, WT = working time, CS = co-worker support, QoL = quality of leadership, JC = job control, SPS = subjective perceived stress, M^U^ = Unconstrained Modell, M = Modell, χ^2^ = = chi-square value, CFI = Comparative Fit Index, RMSEA = Root Mean Square Error of Approximation, SRMR = Standardized Root Mean Squared Residual; Estimation method: ML; **p* < .05. | | | | | | | | | | | | | | | | | | |  | |

**Supplement H Gendered Multi-Group** **SEM Analysis**

***H.1 Male Sample***

| Hypothesized Relationships | | Unstandardized Estimates | | S.E. | | C.R. | | | *p*-values |
| --- | --- | --- | --- | --- | --- | --- | --- | --- | --- |
| QtD ^+^_🡪_ SPS | | 0.33 | | 0.03 | | 11.46 | | | <.001^***^ |
| QlD ^+^_🡪_ SPS | | -0.01 | | 0.04 | | -0.42 | | | .674 |
| WT ^+^_🡪_ SPS | | 0.01 | | 0.03 | | 0.54 | | | .588 |
| CS ^–^_🡪_ SPS | | -0.10 | | 0.03 | | -4.18 | | | <.001^***^ |
| QoL ^–^_🡪_ SPS | | -0.21 | | 0.03 | | -9.35 | | | <.001^***^ |
| JC ^–^_🡪_ SPS | | -0.09 | | 0.03 | | -3.65 | | | <.001^***^ |
|  |  | |  | |  | |  |  | |
| *Note. n* = 2,059. QtD = quantitative demands, QlD = qualitative demands, WT = working time, CS = co-worker support, QoL = quality of leadership, JC = job control, SPS = subjective perceived stress, S.E. = standard error, C.R. = critical ratio; Adjusted for age, educational level, income, children under 14 years in the household, caregiving, hours worked in paid second jobs. Estimation method: ML; **Model Fit Statistics:** χ^2^ = 3648.82, df = 498, CFI = .90, RMSEA = .04, SRMR = .09, AGFI = .90; ***p* < .01. ****p* < .001. | | | | | | | | | |

***H.2 Female Sample***

| Hypothesized Relationships | | Unstandardized Estimates | S.E. | C.R. | *p*-values |
| --- | --- | --- | --- | --- | --- |
| QtD ^+^_🡪_ SPS | | 0.23 | 0.03 | 9.06 | <.001^***^ |
| QlD ^+^_🡪_ SPS | | -0.04 | 0.03 | -1.75 | .080 |
| WT ^+^_🡪_ SPS | | 0.06 | 0.04 | 3.46 | <.001^***^ |
| CS ^–^_🡪_ SPS | | -0.05 | 0.03 | -2.66 | .008^**^ |
| QoL ^–^_🡪_ SPS | | -0.20 | 0.03 | -10.32 | <.001^***^ |
| JC ^–^_🡪_ SPS | | -0.09 | 0.03 | -4.27 | <.001^***^ |
|  |  | | | | |
| *Note. n* = 2,059. QtD = quantitative demands, QlD = qualitative demands, WT = working time, CS = co-worker support, QoL = quality of leadership, JC = job control, SPS = subjective perceived stress, S.E. = standard error, C.R. = critical ratio; Adjusted for age, educational level, income, children under 14 years in the household, caregiving, hours worked in paid second jobs. Estimation method: ML; **Model Fit Statistics:** χ^2^ = 3648.82, df = 498, CFI = .90, RMSEA = .04, SRMR = .09, AGFI = .90; **p* < .05. ***p* < .01. ****p* < .001. | | | | | |
